# Systems-Level Interactome Mapping Reveals Actionable Protein Network Dysregulation Across the Alzheimer’s Disease Spectrum

**DOI:** 10.21203/rs.3.rs-5930673/v1

**Published:** 2025-02-12

**Authors:** Sadik Bay, Anna Rodina, Florence Haut, Tanaya Roychowdhury, Elentina K. Argyrousi, Agnieszka Staniszewski, Kyung Han, Sahil Sharma, Souparna Chakrabarty, Chander S. Digwal, Aleksandra Stanisavljevic, Amanda Labuza, Melissa J. Alldred, Palak Panchal, Anand SanthaSeela, Laura Tuffery, Zhuoning Li, Arsalan Hashmi, Eric Rosiek, Eric Chan, Mara Monetti, Hiroki Sasaguri, Takaomi C. Saido, Julie A. Schneider, David A. Bennett, Paul E. Fraser, Hediye Erdjument-Bromage, Thomas A. Neubert, Stephen D. Ginsberg, Ottavio Arancio, Gabriela Chiosis

**Affiliations:** 1 Chemical Biology Program, Memorial Sloan Kettering Cancer Center, New York, NY 10065, USA.; 2 Taub Institute for Research on Alzheimer’s Disease and the Aging Brain, New York, NY 10032, USA; 3 Tanz Centre for Research in Neurodegenerative Diseases and Department of Medical Biophysics, University of Toronto, Toronto, ON M5R 0A3, Canada; 4 Center for Dementia Research, Nathan Kline Institute, Orangeburg, NY, 10962, USA; 5 Department of Psychiatry, NYU Grossman School of Medicine, New York, NY 10016, USA; 6 Proteomics Core, Memorial Sloan Kettering Cancer Center, New York, NY 10065, USA.; 7 Molecular Cytology Core, Memorial Sloan Kettering Cancer Center, New York, NY 10065, USA.; 8 Laboratory for Proteolytic Neuroscience, RIKEN Brain Science Institute, Wako, Saitama 351-0198, Japan.; 9 Rush Alzheimer’s Disease Center, Rush University Medical Center, Chicago, Illinois 60612; 10 Department of Neuroscience and Physiology, NYU Grossman School of Medicine, New York, NY, 10016, USA.; 11 NYU Neuroscience Institute, NYU Grossman School of Medicine, New York, NY 10016, USA; 12 Department of Medicine, Columbia University, New York, NY 10032, USA; 13 Department of Pathology and Cell Biology, Columbia University, New York, NY 10032, USA; 14 Department of Medicine, Division of Solid Tumors, Memorial Sloan Kettering Cancer Center, New York, NY 10065, USA.

## Abstract

Alzheimer’s disease (AD) progresses as a continuum, from preclinical stages to late-stage cognitive decline, yet the molecular mechanisms driving this progression remain poorly understood. Here, we provide a systems-level map of protein-protein interaction (PPI) network dysfunction across the AD spectrum and uncover epichaperomes—stable scaffolding platforms formed by chaperones and co-factors—as central drivers of this process. Using over 100 human brain specimens, mouse models, and human neurons, we show that epichaperomes emerge early, even in preclinical AD, and progressively disrupt multiple PPI networks critical for synaptic function and neuroplasticity. Glutamatergic neurons, essential for learning and memory, exhibit heightened vulnerability, with their dysfunction driven by protein sequestration into epichaperome scaffolds, independent of changes in protein expression. Notably, pharmacological disruption of epichaperomes with PU-AD restores PPI network integrity and reverses synaptic and cognitive deficits, directly linking epichaperome-driven network dysfunction to AD pathology. These findings establish epichaperomes as key mediators of molecular collapse in AD and identify network-centric intervention strategies as a promising avenue for disease-modifying therapies.

## Introduction

Alzheimer’s disease (AD) is a highly complex and currently untreatable neurodegenerative disorder that unfolds over decades, leading to progressive cognitive decline, primarily affecting memory and executive functions^1^. AD is now recognized as a disease continuum, spanning from preclinical to symptomatic stages, rather than a condition with a discrete onset. Pathophysiological changes begin years before clinical symptoms emerge, in a stage known as preclinical AD^2,3^. This recognition has reframed the study of AD, emphasizing the need to understand the molecular mechanisms driving disease progression across its full spectrum^4^.

Advances in genetics, transcriptomics, metabolomics, and proteomics—coupled with sophisticated neuroimaging—have provided critical insights into AD pathology, revealing widespread molecular and cellular dysregulation and large-scale remodeling of brain networks^5–11^. Yet, despite these breakthroughs, a fundamental gap remains: What drives the progressive molecular collapse of brain networks, and how does this dysfunction evolve over time? Current models, which primarily focus on protein aggregation, amyloid plaques, and tau tangles, fail to fully explain the early molecular events that disrupt cellular networks, ultimately leading to cognitive decline.

To answer these questions, we must look beyond individual disease hallmarks and investigate how protein-protein interaction (PPI) networks are dysregulated in AD. PPIs govern nearly every cellular process^12–14^, and their perturbation is thought to drive key aspects of AD pathology^15,16^. However, mapping these dynamic alterations has remained a major challenge due to the sheer complexity of the interactome and the transient nature of many PPIs^17^. Existing technologies often fail to capture the evolving network dysfunctions in AD, particularly at early disease stages, limiting our ability to identify upstream molecular drivers of neurodegeneration. Addressing this challenge is critical for both understanding AD pathogenesis and identifying therapeutic interventions that can halt or reverse disease progression before irreversible damage occurs.

A systems-level approach is required—one that captures not just static molecular changes but also the dynamic, network-wide reorganization of protein interactions occurring throughout AD. The discovery of epichaperomes, stable disease-associated hetero-oligomeric assemblies of tightly bound chaperones and co-chaperones, offers a crucial breakthrough in this regard^18^. These pathological scaffolding platforms, which emerge specifically under disease conditions^19–23^, actively remodel PPI networks, hijacking cellular pathways that are critical for neuronal function^20,24,25^. By rewiring these networks, epichaperomes drive widespread cellular dysfunction and contribute to the progression of neurodegenerative processes^20,24,25^.

Leveraging this framework, we applied a novel approach to systematically map epichaperome-driven PPI network dysregulation across the AD spectrum. Using a comprehensive collection of over 100 postmortem human brain specimens, combined with experimental validation in AD mouse models and human neurons, we integrated epichaperome biology with our dysfunctional Protein-Protein Interactome (dfPPI) analysis platform^26^ to define how PPI networks are progressively disrupted across AD stages. This approach enables a level of molecular resolution previously unattainable, allowing us to track the evolution of network dysfunction from early preclinical phases to late-stage disease.

Our study provides an unprecedented systems-level map of progressive network dysfunction across the AD continuum, revealing how key cellular pathways are disrupted from early preclinical stages to advanced disease. By integrating epichaperome biology with dfPPI analysis, we uncover a trajectory of molecular dysfunction that begins with the selective vulnerability of glutamatergic neurons and expands to affect broader neuronal circuits and cellular processes over time. Early disruptions are centered on pathways critical for synaptic plasticity and excitatory neurotransmission, including transmission across chemical synapses, glutamatergic synapse signaling, actin remodeling, and long-term potentiation—deficits that manifest well before overt cognitive symptoms emerge. These early perturbations coincide with neuroinflammatory activation and dysregulation of translation initiation and phospholipid metabolism, suggesting a multifaceted molecular cascade that progressively undermines neuronal function. As AD advances, additional network dysfunctions emerge, including impairments in autophagy, iron metabolism, endocytosis, and intracellular trafficking, further exacerbating disease pathology.

Our findings establish epichaperomes as key drivers of these progressive network disruptions. By acting as pathological scaffolding platforms, epichaperomes dynamically rewire protein interactions, leading to maladaptive sequestration of proteins critical for learning and memory, such as Synapsin 1. Notably, the early and selective vulnerability of glutamatergic neurons to epichaperome-driven dysfunction provides a molecular framework for understanding why these neurons are among the first to be affected in AD.

Crucially, we demonstrate that these widespread dysfunctions are not only detectable and mappable but also reversible. Pharmacological disruption of epichaperomes restores PPI network integrity, synaptic function, and cognitive performance in both human neuronal models and APP NL-F mouse models, even at later disease stages. These results challenge the notion that AD-associated dysfunction is irreversible and position network-targeting interventions as a viable therapeutic approach for halting or even reversing cognitive decline.

By systematically dissecting the evolving molecular architecture of AD, this study highlights the central role of network dysfunction in disease pathogenesis and establishes epichaperomes as both a mechanistic driver and an actionable therapeutic target. These findings pave the way for precision medicine strategies aimed at preventing, delaying, or reversing AD-related network dysfunction before irreversible neurodegeneration occurs.

## Results

### Epichaperomes across the AD spectrum

**Figure 1.**
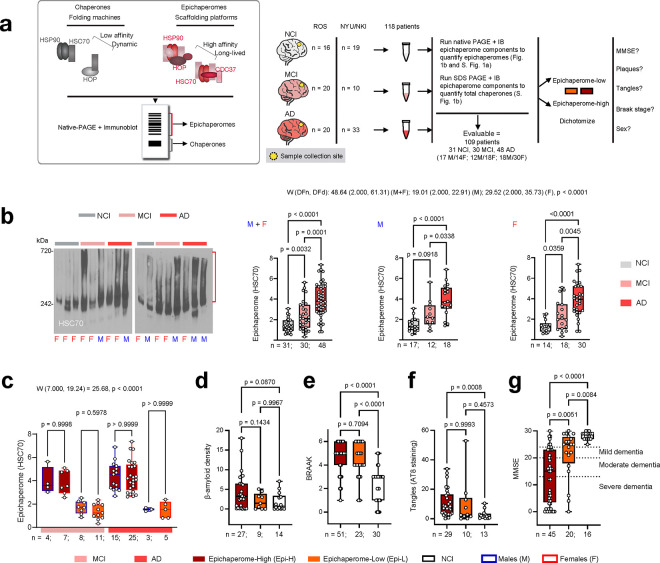
Epichaperome formation emerges early in disease, persists across the AD continuum, and correlates with worse cognitive scores. **a** Experimental design. The cartoon depicts the biochemical distinction between epichaperomes (oligomeric high-molecular weight assemblies with chaperones and co-chaperones nucleating on HSP90 and HSC70) and chaperones (smaller, dynamic assemblies). Due to this distinction, epichaperomes are separated using native PAGE, followed by visualization by immunoblotting with antibodies against epichaperome constituent chaperones (e.g., HSC70). Post-mortem frontal cortex samples from human brains spanning non-cognitively impaired (NCI), mild cognitive impairment (MCI), and Alzheimer’s Disease (AD) stages were assessed for epichaperome content. Patients were then categorized into epichaperome-high and epichaperome-low groups, and their Mini-Mental State Examination (MMSE), Braak stage, tangles, amyloid, and sex were compared across these groups. All data are plotted using a min-to-max box-and-whisker plot, with individual data points representing all values in the dataset. The box indicates the interquartile range, and the line within the box marks the median. Data were analyzed using Brown-Forsythe and Welch ANOVA with Dunnett’s T3 post-hoc test. **b** Epichaperome levels as in **a**, evaluated in NCI, MCI, and AD groups, with data shown for males (M) and females (F) combined, as well as separated by sex. Gel, representative patients profiles of the n = 108 evaluable samples. **c** Epichaperome levels as in **a** in MCI and AD patients divided into low- and high-epichaperome groups, with further separation by sex (male: M, female: F). **d-g** Amyloid (β-amyloid density), W (2.000, 26.69) = 2.720, p = 0.0841 (d), Braak score, W (2.000, 51.30) = 27.70, p < 0.0001 (e), tangles (AT8 staining), W (2.000, 19.95) = 8.456, p = 0.0022 (f), and MMSE scores, W (2.000, 37.31) = 53.22, p < 0.0001 (g), in epichaperome-high (MCI+AD), epichaperome-low (MCI+AD), and NCI groups. Graphs present individual values for each patient in these categories. Source data are provided as Source Data file.

To determine when epichaperomes form along the AD continuum, we initially focused on detecting epichaperomes in the frontal cortex {Brodmann area 9 (BA9)} of postmortem brain specimens from individuals at various stages of cognitive impairment, ranging from non-cognitively impaired aged individuals (NCI) through those with mild cognitive impairment (MCI) to AD dementia. To ensure the robustness and generalizability of our findings, we analyzed samples across multiple patient cohorts (Fig. 1a), incorporating a diverse range of genetic backgrounds, environmental exposures, and clinical manifestations. This approach provided a comprehensive view of epichaperome formation across the AD spectrum, including stages preceding the onset of AD. Details on human postmortem brain specimens and associated variables are found in **Supplementary Data 1,** which contains de-identified patient sample IDs along with assigned variables, including sex, postmortem interval (PMI), age, and clinical covariates such as pathology scores, Mini-Mental State Examination (MMSE), and Braak stage.

Our analysis included 118 patients from two distinct cohorts: the Rush Religious Orders Study (ROS) cohort^27^ (NCI: n=16, 6M/10F, age: 86.1 ± 3.8 years, PMI: 15 ± 8 h; MCI: n=20, 8M/12F, age: 90.3 ± 4.6 years, PMI: 10 ± 6 h; AD: n=20, 7M/13F, age: 90.4 ± 3.4 years, PMI: 11±7 h) and the NYUGSOM/NKI cohort (NCI: n=19, 12M/7F; age: 71.4 ± 17.7 years, PMI: 13 ± 8 h; MCI: n=10, 4M/6F; age: 85.6 ± 5.0 years, PMI: 13 ± 10 h; AD: n=33, 11M/22F; age: 79.0 ± 9.9 years, PMI: 11 ± 5 h). This multi-cohort approach allowed for a thorough understanding of epichaperome dynamics in a vulnerable brain region across various stages and populations of AD.

Epichaperomes, characterized by stable, high-molecular-weight assemblies, are distinct from transient chaperone complexes and can be differentiated using native PAGE separation^20,22,23^. Therefore, to detect and quantify epichaperomes, we employed a validated biochemical method that combines native PAGE with immunoblotting, targeting specific components of epichaperomes such as heat shock protein 90 (HSP90), heat shock cognate protein 70 (HSC70), Hsp70-Hsp90 organising protein (HOP; aka STIP1), and HSP90 co-chaperone cell division cycle 37 (CDC37) (Fig. 1a). We found that while chaperone complexes were consistently present in both normal and diseased brains, epichaperomes were predominantly associated with AD pathology and were detected in the frontal cortex of both MCI and AD patients (Fig. 1b, **Supplementary Fig. 1a**).

We observed that epichaperome levels were significantly elevated as early as the MCI stage (p = 0.0032) and escalated further in AD (NCI to AD, p < 0.0001; MCI to AD, p = 0.0001), a trend consistent across both female and male patients (Fig. 1b). Notably, this increase occurred despite minimal changes in the overall concentration of chaperones across NCI, MCI, and AD groups (**Supplementary Fig. 1a,b**), reinforcing that epichaperome formation is independent of total chaperone protein levels, as reported^21–23,28,29^.

To understand whether differing epichaperome levels correspond to distinct pathological and clinical features, we dichotomized MCI and AD patients into epichaperome-high (Epi-H) and epichaperome-low (Epi-L) groups. The threshold was calculated based on the mean epichaperome levels in the NCI group plus 2 standard deviations, classifying 65.4% of patients as Epi-H and 34.6% as Epi-L (**Supplementary Data 1**). This stratification allowed us to assess whether epichaperome formation associates with specific pathological markers or cognitive decline across the AD spectrum.

We found no significant difference in the distribution of males and females between the Epi-H and Epi-L cohorts, confirming that sex does not influence epichaperome levels (Fig. 1c). We next analyzed whether there was a relationship between epichaperome levels and β-amyloid density (via 4G8, 6F/3D, and 10D immunostaining), Braak stage (tau pathology), phosphorylated tau (via AT8 staining), and MMSE scores (cognitive function)^30,31^ (Fig. 1d–g).

Regarding pathology, our analysis revealed that the Epi-H cohort predominantly included patients with the highest β-amyloid positivity, although this difference did not reach statistical significance (Epi-H vs Epi-L, p = 0.1434; Epi-H vs NCI, p = 0.0870; Fig. 1d). Additionally, we observed significant differences in tau pathology between both the Epi-H and Epi-L groups compared to NCI individuals, as reflected in Braak staging and AT8 levels (Fig. 1e,f, p < 0.0001 for Braak stage in Epi-H vs NCI and Epi-L vs NCI; p = 0.0008 for AT8 in Epi-H vs NCI). However, there was no difference in tau pathology between the Epi-H and Epi-L groups themselves (p = 0.7094 for Braak stage and p = 0.9993 for AT8). Overall, these results imply that epichaperomes may play a significant role in AD independently of direct involvement with amyloid plaques or tau pathology.

On the cognitive front, we found that the higher the epichaperome levels, the more pronounced the cognitive decline (Fig. 1g). Both Epi-H and Epi-L AD and MCI patients exhibited significantly greater impairment than NCI individuals (p = 0.0084 for NCI vs. Epi-L and p < 0.0001 for NCI vs. Epi-H), with Epi-H samples showing significantly greater impairment (lower MMSE scores) than Epi-L samples (p = 0.0051 for Epi-H vs. Epi-L), highlighting a clear link between epichaperome levels and cognitive impairment, with epichaperome formation potentially initiating cognitive decline and driving worsening dysfunction as the disease progresses through the AD continuum.

These results establish that epichaperomes emerge early in AD and increase with disease progression, paralleling cognitive decline. Their presence in the majority of MCI and AD patients suggests they contribute to disease pathogenesis. To determine how epichaperomes drive dysfunction, we next examined their impact on cellular pathways and PPI networks across the AD spectrum.

### Networks dysregulated by epichaperomes across the AD spectrum

**Figure 2.**
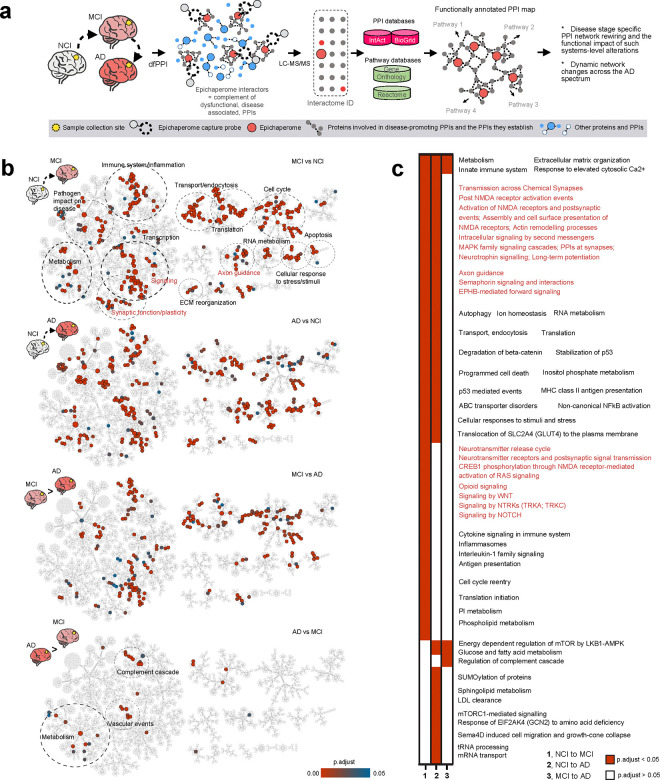
Networks dysregulated by epichaperomes across the AD continuum. **a** Schematic of the experimental design. The diagram illustrates the application of the dysfunctional Protein-Protein Interactome (dfPPI) method to analyze human brain specimens from NYU/NKI cohort including NCI, MCI, and AD, as well as PD samples for comparative control. This panel outlines the chemoproteomic approach used to identify and map the proteins and networks dysregulated by epichaperomes. **b** Reactome mapping of dfPPI results. This panel visualizes the pathways that are differentially enriched or disrupted across the AD continuum, with specific comparisons such as MCI vs. AD, AD vs. NCI and MCI vs. AD highlighted to show distinct shifts in protein enrichment and pathway engagement. **c** Map of dysregulated processes across the AD continuum displays the functional alterations driven by epichaperomes from early stages to late-stage disease. Each row represents a pathway, with major processes selected for representation to manage the complexity of the data. Refer to Supplementary Data 2 for complete datasets and analytics.

To this end, we applied a chemoproteomic method, dysfunctional Protein-Protein Interactome (dfPPI)^26^, to human postmortem brain specimens from the NYUGSOM/NKI cohort {NCI, n = 19; MCI, n = 10; AD, n = 33; and Parkinson’s disease (PD) n = 18} (Fig. 2a). PD samples were included to serve as a comparative disease control, given that epichaperomes also form in PD^32^ but likely dysregulate different cellular processes compared to AD, allowing for the assessment of disease-specific epichaperome-mediated network disruptions.

dfPPI is a powerful interactomic method that uncovers the dynamics of PPIs within the context of epichaperome-mediated perturbations^26^. This approach employs affinity-based probes to capture epichaperomes along with the proteins and protein complexes they sequester in disease states, representing the complement of dysfunctional, disease-associated PPIs in each condition. These captured interactors are then identified and quantified through mass spectrometry, followed by bioinformatic analyses integrating PPI and pathway databases. This enables the derivation of disease stage-specific PPI network rewiring and the functional consequences of such systems-level alterations^26^, including dynamic network changes across the AD spectrum. By revealing how epichaperomes rewire PPIs and alter cellular functions in a disease-specific manner, dfPPI can provide critical insights into the molecular underpinnings of AD. The complete datasets and associated analytics are found in **Supplementary Data 2**.

Across all MCI patients, dfPPI identified 4,214 proteins sequestered by epichaperomes (FC>1 MCI vs. NCI), and 3,998 proteins were similarly sequestered in AD patients (FC>1 AD vs. NCI). Comparison between MCI and AD revealed distinct shifts in protein enrichment, with 2,549 proteins preferentially enriched in AD (FC>1 AD vs. MCI) and 2,923 in MCI (FC>1 MCI vs. AD). Upon applying recommended statistical cutoffs^21^, 1,814 proteins were identified in MCI (MCI vs. NCI) and 2,090 in AD (AD vs. NCI), highlighting widespread network disruptions even at early stages. Importantly, the functional dysregulation caused by epichaperomes was already fully manifest in MCI patients, as indicated by the large number of altered PPIs.

Reactome mapping revealed 403 protein pathways rewired in MCI and 333 in AD (p.adjust < 0.05). The identification of more pathways in MCI underscores the significant functional changes that occur early in the disease, with epichaperome formation driving a large-scale network disruption before the transition to full-blown AD. Comparing MCI and AD, 253 pathways were preferentially enriched in MCI and 38 in AD, further supporting the notion that epichaperomes initiate widespread dysfunction early in the disease, setting the stage for a cascade of changes that continue to unfold as AD progresses. This pattern suggests that while the disease exacerbates some pathways in AD, new pathways are unleashed later in the continuum, reflecting an evolving landscape of cellular dysfunction (Fig. 2b).

Among the earliest epichaperome-mediated disruptions are pathways governing synaptic function and plasticity^33–40^, such as transmission across chemical synapses, glutamatergic synapse, post-NMDA receptor activation events, protein-protein interactions at synapses, actin remodeling, intracellular signaling by second messengers, MAPK family signaling cascades, neurotrophin signaling, long-term potentiation, axon guidance, and the neurotransmitter release cycle (Fig. 2b,c). These pathways are critical for maintaining synaptic communication and stability, processes fundamental to learning and memory, which are significantly impaired in the early stages of AD^41–44^.

Additionally, early epichaperome-driven dysfunction extends to neuroinflammatory responses, including pathways involved in cytokine signaling, inflammasomes, interleukin-1 family signaling, and antigen presentation (Fig. 2b,c), reflecting an early unleashing of inflammatory processes that contribute to neuronal damage^45–49^. These inflammatory responses likely play a key role in the early neuronal dysfunction observed in MCI, even before overt cognitive decline is evident.

Processes related to cell cycle reentry, translation initiation, and phospholipid metabolism are also dysregulated by epichaperomes at the MCI stage (Fig. 2b,c). This suggests that epichaperome formation in neurons may inappropriately push them to re-enter the cell cycle, disrupt protein synthesis, and ultimately compromise neuronal function^50,51^. As the disease progresses, epichaperome-mediated dysregulation of autophagy, iron metabolism, trafficking, and endocytosis becomes more pronounced (Fig. 2b,c), leading to impaired cellular waste removal and nutrient recycling, and contributing to the buildup of toxic proteins^52–54^.

In AD, the reliance of metabolic, vascular, and complement cascade pathways^55–57^ on epichaperomes becomes increasingly evident (Fig. 2b,c), reflecting their pivotal role in driving systemic dysfunction that exacerbates neuronal damage and accelerates cognitive decline. The epichaperome-mediated dysregulation of complement cascade processes further suggests that a sustained and detrimental inflammatory response persists throughout the AD continuum, driven by the aberrant scaffolding activity of epichaperomes.

In summary, these results highlight that epichaperomes begin to disrupt critical brain networks early in the AD spectrum with widespread functional alterations already fully manifest in MCI. These changes continue to cascade as the disease progresses, impairing essential neuronal processes and contributing to systemic dysfunction. The evolving nature of these network disruptions underscores the pivotal role of epichaperomes in reshaping brain function, driving both early and late-stage AD pathology.

### Trajectory of epichaperome formation in AD

**Figure 3.**
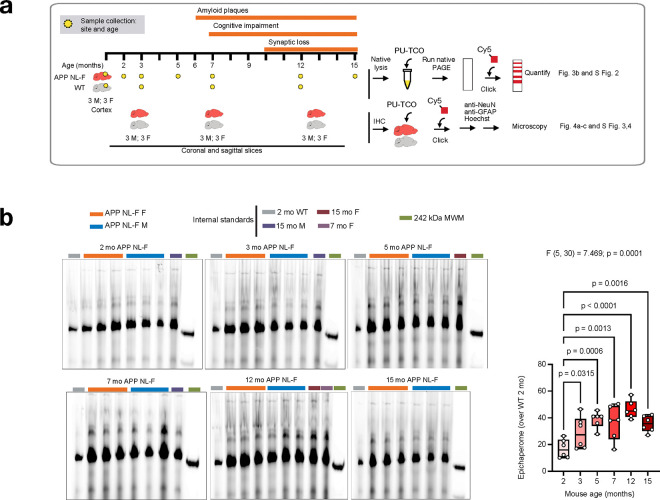
Epichaperome formation begins in the preclinical stages of Alzheimer’s disease. **a** Study design to assess the spatiotemporal trajectory of epichaperome formation in AD-vulnerable brain regions of APP NL-F mice compared to control (WT) mice, with the aim of identifying anatomical and cellular vulnerability to epichaperome formation over time. Epichaperome levels were analyzed both in the cortex (via native PAGE separation and detection with PU-TCO clicked to Cy5 dye) and across the whole brain (using confocal microscopy on sagittal and coronal slices stained with PU-TCO clicked to Cy5 dye) at targeted age intervals. For native PAGE and immunodetection analysis, assessments were conducted at 2, 3, 5, 7, 12, and 15 months of age, with 3 males and 3 females per group, focusing on cortical regions associated with cognitive and synaptic vulnerability in AD. For microscopy-based analyses, coronal sections representing key AD-vulnerable regions (dentate gyrus, CA1, CA3, dorsal subiculum, entorhinal cortex, and frontal cortex) were examined at 3, 7, and 12–14 months of age in 3 male and 3 female mice per group. This design allowed for a detailed analysis of the spatiotemporal progression and regional specificity of epichaperome formation across AD-relevant stages in APP NL-F mice. **b** Trajectory of epichaperome levels in the APP NL-F mouse cortex across disease stages, evaluated via native PAGE separation and detection with PU-TCO clicked to Cy5 dye. See Supplementary Fig. 2 for WT mice. Data are plotted using a min-to-max box-and-whisker plot, with data points representing individual mice. The box indicates the interquartile range, and the line within the box marks the median. Data were analyzed using one-way ANOVA followed by the Two-stage linear step-up procedure of Benjamini, Krieger, and Yekutieli for multiple comparisons, with p < 0.05 considered significant. Source data are provided as a Source Data file.

Given that widespread epichaperome-mediated dysfunctions are already manifest in the MCI stage, this raises the question of how early in the disease these disruptions begin. While our human patient samples cover MCI and AD stages, they do not include preclinical AD, a phase where significant brain changes occur prior to clinical symptom onset. The systems-level analysis in MCI revealed that epichaperomes are already driving widespread dysfunction at this early stage, strongly indicating that epichaperome formation may begin even earlier—during preclinical AD. To investigate these earliest stages of epichaperome formation, we utilized the APP NL-F mouse model, which replicates the full spectrum of AD, from preclinical stages to late-stage disease^58^. We conducted a detailed analysis of epichaperome formation at various ages (2, 3, 5, 7, 12, and 15 months) to encompass the full disease trajectory. As controls, we used C57BL/6J (wild-type, WT) mice (Fig. 3a).

In APP NL-F mice, amyloid plaques composed of Aβ42 begin forming around 6 months of age^59^, but significant changes in brain function, such as hypersynchrony—excessive synchronized activity between brain regions—emerge as early as 3 months^60^. This hypersynchrony is most prominent in the hippocampal and frontal/cingulate networks and parallels neural disruptions observed in preclinical AD. These early changes occur well before synaptic loss, which typically manifests between 9 and 12 months^59^. Cognitive flexibility impairments are evident by 3 months, followed by spatial memory deficits at 7 months, representing the transition from preclinical AD to MCI and later symptomatic stages^58^. The APP NL-F model, therefore, enables investigation of epichaperome formation across all stages, including preclinical changes.

By combining native PAGE separation with blotting using PU-TCO, an epichaperome-specific small molecule probe^32^, we detected epichaperome formation in the cortex of APP NL-F mice as early as 2–3 months (Fig. 3b)—well before the onset of amyloid plaque formation (reported at 6 months)^59^ and prior to the emergence of spatial memory deficits (reported at 7–8 months)^58^. Epichaperome levels increased gradually, peaking between 5–7 months and remaining high thereafter (Fig. 3b). Similar to the human condition, epichaperomes were predominantly present in APP NL-F mice compared to WT controls and their presence was independent of chaperone concentration in the cortex (**Supplementary Fig. 2a,b**).

**Figure 4.**
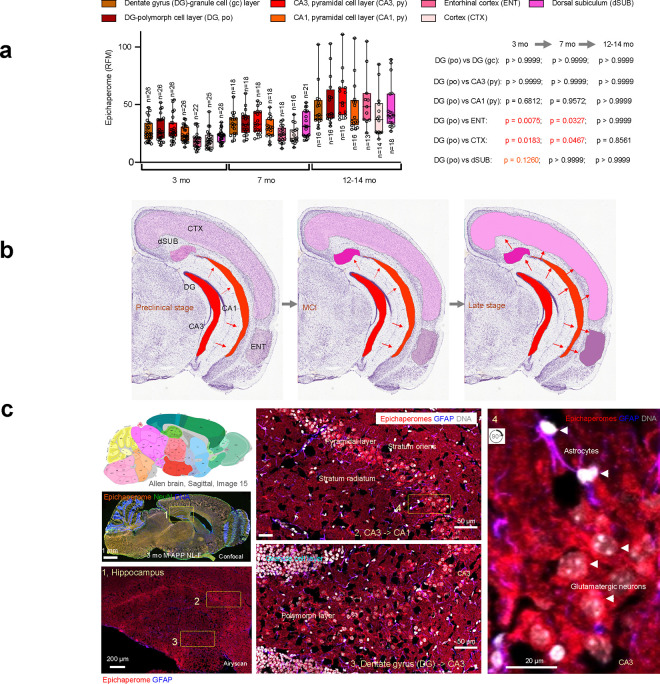
Epichaperomes form in key AD-vulnerable brain cells and regions, progressively increasing in levels and spatial distribution as disease advances in APP NL-F mice. **a** Epichaperome quantification in APP NL-F mice at 3, 7, and 12–14 months of age (3 females and 3 males per group) highlights their early formation and progressive accumulation in AD-vulnerable brain regions. Coronal brain slices stained with PU-TCO clicked to Cy5 dye, corresponding to Bregma −1.22 mm to −2.54 mm (Allen Brain Atlas, images 80–87), were analyzed across regions associated with memory and cognitive function. Data are presented with box and whiskers indicating the minimum to maximum range, with the interquartile range boxed and the median line indicated. Each data point represents an individual brain slice. Statistical analysis among regions within each age group was performed using Brown-Forsythe and Welch ANOVA (F*(DFn, DFd) = 12.56 (20.00, 160.3); p < 0.0001) with Dunnett’s post-hoc test. See also Supplementary Fig. 4,5. **b** Schematic illustration of the spatiotemporal trajectory of epichaperome formation in APP NL-F mice, showing highest early levels in the CA3 and dentate gyrus at 3 months, with CA1 following in intensity and dorsal subiculum, entorhinal cortex, and frontal cortex subsequently affected. Levels in all regions progressively increase by 7 months, with widespread, equally high levels across all regions by 12–14 months, suggesting a gradual progression from initial epichaperome formation in CA3 and DG toward CA1, dorsal subiculum, entorhinal cortex, and eventually the frontal cortex. Figure adapted using Allen brain, coronal slice image 85. **c** Representative sagittal slice of an APP NL-F mouse brain as in a stained with PU-TCO clicked to Cy5 dye, showing epichaperome formation. Inset 1 highlights the hippocampus, revealing strong epichaperome staining in the dentate gyrus (DG) and CA3-CA1 regions. Zoomed-in regions 2–4 show intense signal in glutamatergic neurons and surrounding astrocytes. GFAP marks astrocytes, NeuN labels neurons, and Hoechst marks nuclei. Source data are provided as a Source Data file.

To further confirm epichaperome formation during the preclinical stage of AD and investigate the specific cellular vulnerabilities to epichaperome formation, we performed click chemistry labeling on brain slices using PU-TCO (epichaperome probe) and PU-NTCO (control probe) (Fig. 3a, 4a–c and **Supplementary Figs. 3,4**)^32^. Slices were counterstained with Hoechst to visualize individual cells, and immunostaining for NeuN and glial fibrillary acidic protein (GFAP) distinguished neurons from astrocytes. WT mice, as well as slices pre-treated with the epichaperome disruptor PU-H71, served as negative controls^18,61^, with no detectable signal in these conditions, confirming probe specificity (**Supplementary Fig. 3,4**).

We analyzed epichaperome formation in key AD-vulnerable brain regions in APP NL-F mice at 3, 7, and 12–14 months of age, focusing on hippocampal subfields (pyramidal layers of CA1 and CA3, the polymorph and granule cell layers of the dentate gyrus, DG), dorsal subiculum, entorhinal cortex, and frontal cortex (Fig. 4a and **Supplementary Fig. 5**). These regions, associated with synaptic plasticity, spatial navigation, contextual memory, and executive functions, represent key circuits progressively affected in AD^62,63^.

At 3 months, epichaperome signal was already detectable, with the strongest signal observed in CA3 and the DG, followed closely by CA1, then dorsal subiculum, entorhinal cortex, and frontal cortex (Fig. 4a). Quantitative analysis revealed no significant difference between CA3 and DG. Signal in CA1 was slightly lower but not significantly different compared to the DG, while epichaperome levels in hippocampal regions (CA3, DG) were significantly higher than in subiculum and cortical regions (e.g., DG vs. entorhinal cortex, p = 0.0075; DG vs. frontal cortex, p = 0.0183). WT mice showed no detectable signal in any region (DG APP vs. WT, p < 0.0001) (**Supplementary Fig. 4**).

By 7 months, epichaperome levels had increased across all brain regions analyzed, with the highest levels in CA3, CA1, and DG. The difference among these hippocampal subfields (CA3, DG, and CA1) and the dorsal subiculum was no longer observed (p values were > 0.9999), indicating a progressive spread within hippocampal formation subfields. Despite this convergence in hippocampal subfields, significant differences remained between hippocampal and cortical regions, with DG levels still significantly higher than both entorhinal cortex (p = 0.0053) and frontal cortex (p = 0.0063). WT mice continued to show no detectable signal in any region, reinforcing that epichaperome formation is specific to the AD disease progression observed in APP NL-F mice.

At 14 months, epichaperome levels reached comparably high intensities across all regions, with no significant differences among them (p values > 0.9999 across comparisons). WT mice again showed no detectable epichaperome signal (**Supplementary Fig. 4**).

These findings demonstrate that epichaperome formation follows a distinct spatiotemporal trajectory in AD-vulnerable regions (Fig. 4b) paralleling findings in human AD^62,63^. Initial high levels are detected in CA3 and DG at the early preclinical stage (3 months), followed closely by CA1, then dorsal subiculum, entorhinal cortex, and finally the frontal cortex. This progression suggests that epichaperome formation initiates in DG/CA3 and gradually spreads, involving sequentially larger brain areas as the disease advances, with a continuous increase in levels across all regions by late-stage disease (12–14 months).

Notably, these earliest vulnerable regions—CA3, DG, and CA1—are primarily composed of excitatory neurons, particularly glutamatergic neurons, which are critical for synaptic plasticity and memory^35^. Indeed, in terms of earliest cellular vulnerability, high-resolution imaging of the 3-month-old APP NL-F hippocampus revealed pronounced epichaperome formation in glutamatergic neurons and adjacent astrocytes, affecting soma, nuclei, and projections at early stages (see inset showing glutamatergic neurons in CA3, Fig. 4c). This suggests a region-specific susceptibility rooted in the cellular composition, with epichaperome formation targeting glutamatergic neurons and their functionally supportive glia, potentially disrupting the synaptic plasticity and cognitive functions associated with these regions at the earliest stages of AD onset.

In sum, our analysis of the APP NL-F mouse model revealed that epichaperome formation begins in the early, preclinical stages of AD, well before the onset of amyloid plaque formation and cognitive decline. Epichaperomes were detectable as early as 2–3 months (preclinical stage), with levels peaking between 5–7 months (MCI stage), particularly in hippocampal subfields known for their excitatory, glutamatergic composition. By the late-stage disease (12–14 months), epichaperome levels reached comparably high intensities across all brain regions analyzed, indicating a widespread and homogenized pattern of epichaperome formation. This region- and cell-type-specific vulnerability underscores the selective impact of epichaperomes on key brain areas essential for memory and synaptic function, positioning glutamatergic neurons as pivotal drivers of early network disruptions. The continuous expansion and escalation of epichaperome formation across interconnected brain regions support a model in which epichaperomes contribute to the structural and functional progression of AD, ultimately driving widespread network dysfunction by late-stage disease.

### Glutamatergic neurons vulnerability to epichaperome formation

**Figure 5.**
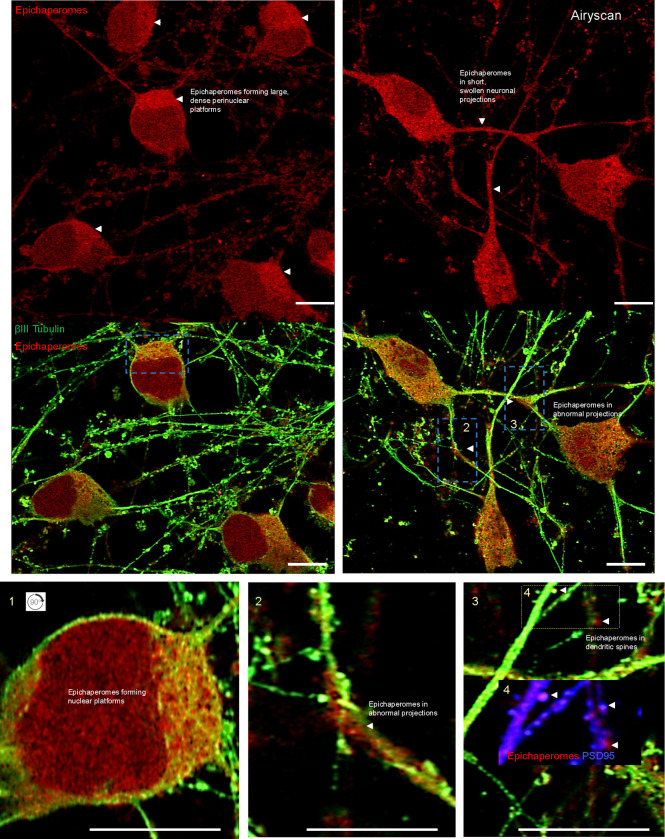
Glutamatergic neurons form epichaperomes under Alzheimer’s disease-related stressors. Micrographs display human iCell Glutamatergic Neurons (iGluts) treated with oligomeric Aβ42, highlighting the susceptibility of these neurons to epichaperome formation under Alzheimer’s disease-related stressors. The main images show widespread epichaperome presence throughout the neuronal soma and projections. Inset 1 emphasizes large perinuclear epichaperome platforms as well as the formation of nuclear platforms. Zoomed-in regions 2 and 3 detail epichaperome signals within axonal swellings and dendritic spines, respectively. Region 4, an additional zoom of region 3, is co-stained with PSD95 to further highlight the localization of epichaperomes in dendritic spines. Epichaperomes are marked by PU-TCO clicked to cy5 (red), neurons by anti-betaIII tubulin (green), and PSD95 is shown in blue. See also Supplementary Fig. 3 and 4. Micrographs are representative of 50 neurons from three independent experiments. Scale bars, 5 μm.

Given the early formation of epichaperomes in glutamatergic neurons observed in the APP NL-F mouse model, we sought to determine whether human neurons display a similar vulnerability under AD-related stress. To investigate this, we used human induced pluripotent stem cell-derived glutamatergic neurons (iGlut) to explore epichaperome formation under disease-relevant conditions (Fig. 5, **Supplementary Fig. 6a-c**). We confirmed that iGlut neurons, when derived from healthy patients, exhibited minimal to no epichaperome levels under normal conditions, commensurate with epichaperome formation being a feature specific to disease^18,24^ (**Supplementary Fig. 6a-c**).

We had previously shown that application of oligomeric Aβ42 (oAβ42), a well-established AD-related stressor^64,65^, causes significant alterations in glutamatergic neurons, including synaptic protein redistribution, such as Synapsin 1, as well as cytoskeletal disruptions^66^. These changes impair synaptic plasticity and alter neurotransmitter release^66^, making this model ideal for further exploration of epichaperome formation. Indeed, we found that treatment with oAβ42, but not scrambled Aβ peptide, robustly induced epichaperome formation in iGlut neurons (Fig. 5, **Supplementary Fig. 6a-c**). This was corroborated in another cellular model, N2a neuronal cells, where similar results were observed (**Supplementary Fig. 7a-c**).

Epichaperomes formed large platforms within the soma, spanning both the cytosolic and nuclear regions, with pronounced perinuclear formations. Additionally, epichaperomes were present in neuronal projections, including within dendritic spines (Fig. 5). Notably, in projections exhibiting morphological abnormalities—specifically, being thicker and shorter than typical—epichaperomes accumulated at sites of swelling. These sites also displayed disrupted cytoskeletal structure as indicated by altered βIII tubulin staining, a hallmark contributing to synaptic dysfunction and impaired connectivity in various neurodegenerative conditions, including AD and PD^67,68^.

In conclusion, these cell-based findings confirm that glutamatergic neurons are particularly susceptible to epichaperome formation under AD-related stress. The extensive formation of epichaperomes throughout the neuronal soma and projections underscores the vulnerability of these neurons, with significant implications for synaptic function and neuronal integrity. The accumulation of epichaperomes in regions essential for neurotransmission, such as dendritic spines and axonal swellings, strongly supporting a role in the synaptic dysfunction observed in AD.

### Synaptic function remodeling across the AD continuum

**Figure 6.**
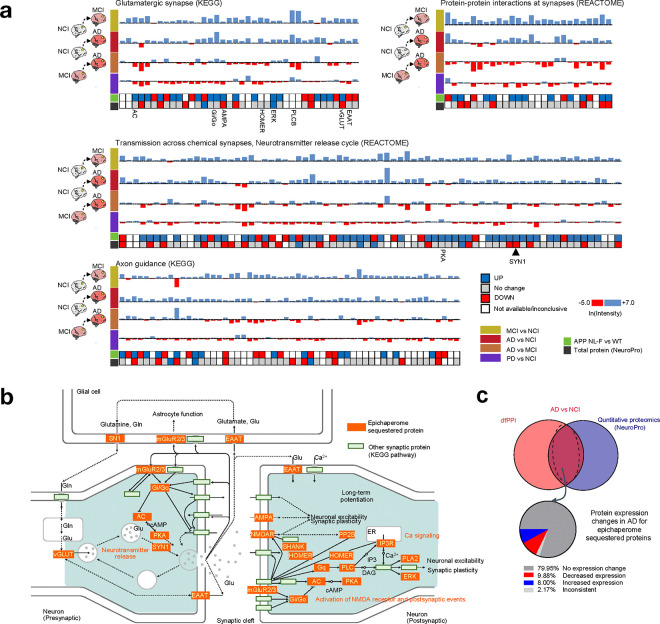
Impact of epichaperomes on synaptic function and plasticity across the AD continuum. **a** Changes in specific proteins within pathways related to synaptic function and plasticity across the AD continuum, based on dysfunctional Protein-Protein Interactome (dfPPI) analysis. Blue bars represent proteins sequestered by epichaperomes in one comparison, while red bars indicate proteins that are less sequestered or not captured in subsequent stages, showing how the composition of proteins affected by epichaperomes evolves as the disease progresses from mild cognitive impairment through advanced stages of AD. dfPPI detected changes in PD and APP NL-F mice (15 mo F) and protein expression changes in AD detected by quantitative proteomics in bulk brain tissue (NeuroPro database) are shown for comparison. **b** Adapted KEGG glutamatergic synapse pathway illustrates synaptic proteins impacted by epichaperomes across the AD continuum. **c** The Venn diagram compares proteins identified by dfPPI as sequestered by epichaperomes in AD versus changes in protein expression in AD detected by quantitative proteomics in bulk brain tissue (NeuroPro database). This comparison highlights that protein sequestration into epichaperomes occurs independently of overall expression changes. See Supplementary Data 3,4 for complete datasets and analytics.

Building on earlier findings of epichaperome formation in glutamatergic neurons and their involvement in synaptic dysfunction, we focused on how epichaperomes impact key glutamatergic neuron-dependent pathways related to synaptic function and plasticity across the AD spectrum (Fig. 6a–c and **Supplementary Data 3**). These dfPPI-identified pathways—such as transmission across chemical synapses, glutamatergic synapses, PPIs at synapses, and axon guidance (Fig. 6a,b)—are critical for neuron-to-neuron communication and the maintenance of cognitive functions, particularly learning and memory, which are heavily reliant on glutamatergic neurotransmission^69^.

Our analysis of the dfPPI data revealed that proteins involved in these critical synaptic processes were sequestered by epichaperomes (depicted by blue bars, Fig. 6a) early in the disease, during the MCI stage (see MCI vs. NCI), and remained sequestered as the disease progressed (see MCI vs. NCI and AD vs. MCI, blue bars). Interestingly, we observed dynamic changes as AD advanced, with new proteins being recruited to epichaperomes (blue bars) and others excluded (red bars). This dynamic remodeling suggests that the synaptic dysfunction observed early in the disease is not static but evolves as epichaperomes continuously reorganize protein networks, and that epichaperome-mediated remodeling of synaptic networks is an evolving process, continually reshaping protein interactions. Importantly, these changes were AD-specific, as the patterns of protein sequestration and exclusion differed when comparing AD and PD (Fig. 6a, see AD vs. NCI compared to PD vs. NCI), supporting the idea that epichaperome-driven disruption manifests differently functionally across distinct neurodegenerative disorders.

A crucial finding from our study is that the sequestration of proteins into epichaperomes occurred independently of changes in protein expression levels. When comparing the proteins identified by dfPPI as sequestered by epichaperomes to those detected by quantitative proteomics in bulk brain tissue (total protein, NeuroPro database)^70^, we found that the expression of epichaperome-sequestered proteins remained unchanged in AD for the majority (79.95% of proteins), with only a small fraction showing changes in concentration (9.88% with decreased levels and 8% with increased levels in AD vs. NCI) (see NeuroPro data versus dfPPI data, Fig. 6a,c and **Supplementary Data 4**). This stands in contrast to the classical view that changes in protein expression are key drivers of disease. Here, it is the sequestration into epichaperome platforms, rewiring their local interactome, that drives functional disruption.

To further support this notion, we focused on Synapsin 1 (SYN1)—a key synaptic protein that we identified as an epichaperome interactor in AD (**Supplementary Data 2**). Previous studies have shown that Synapsin 1 levels remain either unchanged or slightly decreased in AD brains and in cellular and mouse models of AD^70–72^. Despite these minimal changes in expression, Synapsin 1 mislocalization is often observed in these disease models and is directly linked to Synapsin dysfunction. This mislocalization affects axonal transport, reduces synaptic vesicle availability at presynaptic terminals, and disrupts neurotransmitter release, ultimately impairing synaptic plasticity and memory^68,73,74^.

**Figure 7.**
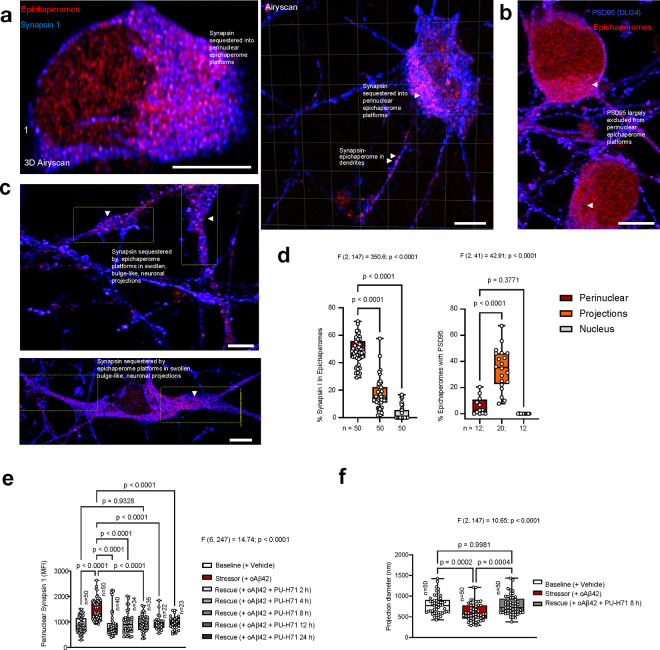
Epichaperomes sequester Synapsin 1 altering its physiological cellular location and distribution. **a,b** Micrographs - representative of 50 neurons from three independent experiments - show human iCell Glutamatergic Neurons (iGlut neurons) treated with oligomeric Aβ42 (100 nM, 24 h), highlighting Synapsin 1 sequestration by epichaperomes into perinuclear platforms (a) and at aberrant neuronal projection sites (b). **c** PSD95, another important synaptic protein, is largely excluded from the large perinuclear epichaperome platforms. Epichaperomes are marked by PU-TCO clicked to Cy5 (red), while Synapsin 1 and PSD95 are shown in blue. Scale bars represent 5 μm. **d** Colocalization analysis of epichaperomes with Synapsin 1 and PSD95 in the specified neuronal regions, indicating their spatial association across these cellular regions. **e,f** Rescue: iGlut neurons were exposed to stressor (100 nM oligomeric Aβ42, 24 h) followed by treatment with an epichaperome disruptor (1 μM PU-H71, 2 to 24 h). **d-f** All data are plotted using a min-to-max box-and-whisker plot, where individual data points represent analyzed regions across 50 neurons for Synapsin 1 and 12 for PSD95 (d) and individual neurons for (e) and individual projections for (f) from 3 independent experiments. The box indicates the interquartile range, and the line within the box marks the median. Data were analyzed using one-way ANOVA with Sidak’s post-hoc test. See also Supplementary Fig. 9. Source data are provided as a Source Data file.

Using iGlut neurons under oAβ42 stress and high-resolution microscopy (Airyscan), we confirmed that this stressor does indeed lead to Synapsin 1 mislocalization without significantly altering Synapsin 1 expression (**Supplementary Fig. 8a-c**). Under normal physiological conditions, Synapsin 1 is typically localized at presynaptic terminals, where it binds to synaptic vesicles and helps maintain them in the reserve pool, controlling their availability for release during synaptic transmission^74,75^. However, in neurons exposed to stress, we observed a markedly different distribution: Synapsin 1 was found primarily in the perinuclear region, rather than at presynaptic terminals, and was largely associated with epichaperome platforms at this location (Fig. 7a and **Supplementary Fig. 8a-c,9a,b**). Smaller, focal formations of epichaperomes and Synapsin 1 were also observed throughout axons and dendrites (Fig. 7a,b). Notably, neurons displaying altered projection morphology—characterized by shorter, thicker structures—showed increased Synapsin 1 sequestration into epichaperomes at the base of these projections and along swollen or bulge-like structures (Fig. 7b). Not all synaptic proteins showed similar epichaperome sequestration, as for example PSD95 (aka DLG4), was largely excluded from perinuclear platforms (Fig. 7c). Crucially, treatment with the epichaperome disruptor PU-AD restored Synapsin 1 distribution (Fig. 7d and **Supplementary Fig. 9a,b**) to normal levels (baseline vs 8 h PU-H71, p = 0.9328), demonstrating that the negative impact of epichaperome sequestration of Synapsin 1 manifests, in part, through altered physiological cellular localization. This restoration was accompanied by a reversal of projection morphology abnormalities, with neuronal projections regaining baseline-like structural integrity (Fig. 7e and **Supplementary Fig. 9a,b**).

In summary, these synaptic interrogation findings indicate that epichaperome formation significantly impacts pathways related to synaptic function and plasticity early in AD and continues to remodel these pathways as the disease progresses. Proteins involved in these pathways, such as Synapsin 1, are sequestered by epichaperomes, leading to their altered distribution, which may impact function despite no changes in cellular protein concentration. This underscores the vulnerability of glutamatergic neurons to epichaperome formation and the continuous disruption of synaptic processes throughout the disease continuum. While a detailed molecular understanding of this sequestration is beyond the scope of this study, these results link prior findings of synaptic protein mislocalization in stressed neurons to a mechanism: sequestration into epichaperome platforms as a root cause of mislocalization.

### Trajectory of epichaperome-mediated synaptic dysfunction

**Figure 8.**
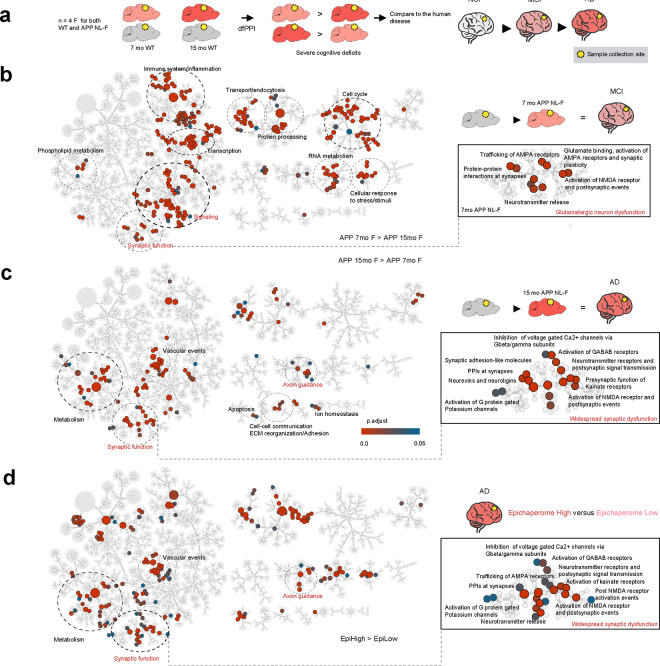
Temporal trajectory of synaptic networks dysregulated by epichaperomes in APP NL-F mice and human AD brains. **a** Schematic of the experimental design. The diagram illustrates the application of the dysfunctional Protein-Protein Interactome (dfPPI) method to analyze brain specimens from APP NL-F mice at 7 months (early-stage cognitive decline) and 15 months (late-stage cognitive decline), as well as age-matched wild-type (WT) controls for comparison. **b,c** Reactome pathway analysis of dfPPI results in APP NL-F mice. The maps display the pathways dysrupted across the disease continuum, with **b** showing the shift in pathways between 7 and 15 months of age (APP 7mo F > APP 15mo F, reflecting changes in the MCI human disease) and **c** highlighting late-stage changes (APP 15mo F > APP 7mo F, reflecting changes observed in late-stage human AD). Specific synaptic pathways related to glutamatergic dysfunction in early stages (7mo) and additional disruptions involving inhibitory circuits, such as GABAergic signaling, in later stages (15mo) are highlighted. **d** Similar analysis in human AD patients, comparing epichaperome-high (EpiHigh) vs. epichaperome-low (EpiLow) cohorts. These results show broader synaptic dysfunction across multiple neuronal subtypes in patients with elevated epichaperome levels, underscoring the progressive nature of epichaperome-mediated network dysfunction in AD. Refer to Supplementary Data 5,6 for complete datasets and analytics.

Given the evolving impact of epichaperome formation on glutamatergic synaptic function, we next sought to better delineate the linear trajectory of these synaptic disruptions as the disease progresses. Specifically, we aimed to determine whether the increasing levels of epichaperomes observed throughout AD correlate with a broadening impact on additional synaptic pathways and neuronal types beyond glutamatergic neurons. To explore this, we applied dfPPI to female APP NL-F mice at 7 months of age—when cognitive deficits begin to appear—and 15 months of age—when cognitive deficits become more severe—along with age-matched wild-type (WT) controls (Fig. 8a and **Supplementary Data 5**).

We found that protein network dysfunctions in APP NL-F mice closely mirrored patterns observed in human AD brains. At 7 months, these mice displayed dysfunctions across a range of pathways that are also disrupted in early-stage human AD, including signaling, inflammatory responses, cell cycle re-entry, transcription, RNA metabolism, and stress responses (Fig. 8b). Notably, synaptic dysfunction was akin to that seen in human MCI disease, with disruptions in glutamatergic neuronal activities, such as NMDA receptor activation, neurotransmitter release, AMPA receptor signaling, synaptic plasticity, and synaptic protein interactions (Fig. 8b). Metabolic dysfunction was primarily in phospholipid metabolism, and thus less pronounced compared to human disease.

By 15 months, additional dysfunctions were evident, closely aligning with late-stage human AD. This included disruptions in metabolic pathways, vascular events, axon guidance, cellular adhesion, and extracellular matrix organization (Fig. 8c). There was a significant shift from early glutamatergic dysfunction to broader disruptions of both excitatory and inhibitory circuits and presynaptic mechanisms. Newly affected pathways included GABAergic signaling, kainate receptor activity, G protein-gated potassium channels, and neurexin/neuroligin signaling, highlighting the disease’s impact on inhibitory circuits and presynaptic processes (Fig. 8c)^76–79^. These changes suggest a broader neuromodulatory dysregulation, affecting calcium homeostasis and synaptic architecture, crucial for maintaining neuronal communication and stability.

In summary, the transition from 7 to 15 months reflects a shift from early-stage glutamatergic dysfunction to broader synaptic and neuronal network disruptions, involving both excitatory and inhibitory pathways. This suggests that as the disease progresses, and epichaperomes expand their negative impact, not only glutamatergic neurons but also other neuronal subtypes become affected by epichaperomes, leading to widespread brain network failure.

We confirmed this progression in human AD patients. Similar to the broader synaptic dysfunctions observed in the 15-month-old APP NL-F mouse brains, patients with higher epichaperome levels (Epi-H) exhibited a broader spectrum of disrupted synaptic functions compared to those with lower epichaperome levels (Epi-L) (Fig. 8d and **Supplementary Data 6**). In Epi-H patients, the disruption extended beyond the glutamatergic networks observed in earlier disease stages, encompassing additional synaptic pathways and involving multiple neuronal subtypes, much like the progression seen in the APP NL-F model at 15 months.

This indicates that as epichaperome levels increase, an expanding set of synaptic pathways and neuronal types become progressively affected. The progression from initial glutamatergic dysfunctions to broader synaptic disruption highlights the dynamic and expanding influence of epichaperomes on brain networks. The data demonstrate that worsening cognitive symptoms in AD are closely linked to the spread and intensification of epichaperome activity. As epichaperome levels rise and these platforms extend to additional cells and brain regions, they progressively co-opt new synaptic networks, further accelerating cognitive decline and exacerbating neuronal dysfunction.

### Validation of epichaperome-mediated dysfunctions

Our postmortem human studies identified numerous protein networks dysregulated by epichaperomes across the AD spectrum particularly those related to synaptic function, neuroplasticity, and memory. To directly link these disruptions to epichaperome formation and determine whether they are reversible, we turned to the APP NL-F mouse model. This model is ideal for two key reasons: first, the same synaptic pathways disrupted by epichaperomes in human AD were also identified in APP NL-F mice through dfPPI, establishing a consistent link between epichaperome formation and synaptic dysfunction in both species. Second, the glutamatergic neurons in the hippocampus—responsible for executing these synaptic functions—were found to be particularly vulnerable to epichaperome formation early in disease progression, a pattern mirrored in human AD.

These early epichaperome formations target glutamatergic neurons, particularly affecting excitatory synapses and disrupting synaptic plasticity and cognitive functions. This selective vulnerability underlies early-stage deficits in memory and learning, driven by disruptions in key pathways such as those involved in long-term potentiation (LTP) and neurotransmitter release. As the disease progresses, epichaperomes expand beyond glutamatergic neurons, sequestering additional synaptic proteins and disrupting other neuronal types, including GABAergic neurons and inhibitory circuits. This progression culminates in broader disruptions of synaptic networks, as seen in the dfPPI analysis of 15-month-old APP NL-F mice, where both excitatory and inhibitory pathways are compromised.

**Figure 9.**
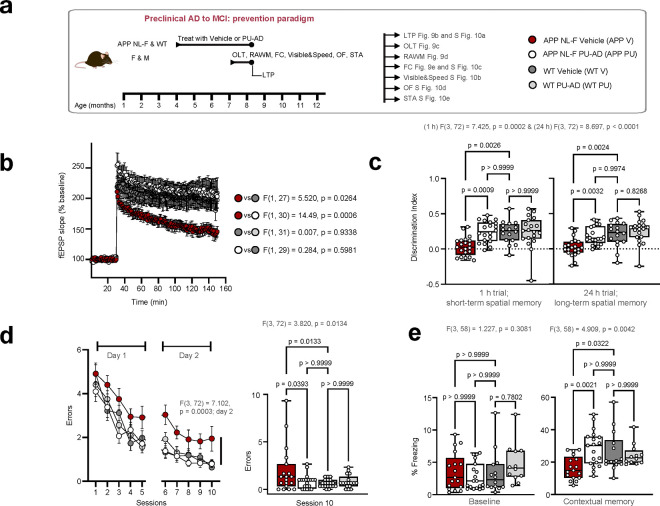
PU-AD treatment prevents synaptic plasticity and memory deficits in APP NL-F mice. **a** Schematic of the experimental design for APP NL-F (APP) and WT littermates treated with vehicle (V) or PU-AD (PU) as indicated. Treatment started at an age where the mice do not exhibit deficits (4 months of age), and continued for 3 months, until an age when the mice display moderate impairments (4 months of age). OLT, object location task; RAWM, radial arm water maze; FC, fear conditioning; OF, open field; STA, sensory threshold assessment; LTP, long-term potentiation. **b** LTP as in **a** measured as fEPSP slope (% baseline) over time in hippocampal slices from individual mice. LTP measures synaptic plasticity, with higher fEPSP slopes indicating stronger synaptic responses. Graph, mean ± s.e.m., two-way repeated measures (RM) ANOVA. Slices: n = 15 (3M,5F) for APP V; n = 17 (4M,3F) for APP PU; n = 14 (4M,5F) for WT V; and n = 19 (5M,6F) for WT PU. **c** Discrimination index (DI) in the OLT reflects the ability of individual mice to distinguish between moved and non-moved objects. Short-term spatial memory was determined as in **a** with a 1-hour interval between the learning and the test trial, and long-term spatial memory with a 24-hour interval between trials. Higher DI indicates better spatial memory. Mean ± s.e.m., one-way ANOVA with Bonferroni’s post-hoc, n = 21 (11M,10F) for APP V; n = 20 (11M,9F) for APP PU; n = 16 (9M,7F) for WT V; n = 19 (9M,10F) for WT PU. **d** RAWM performance, shown as mean ± s.e.m. errors made over sessions, evaluates short-term reference memory and spatial learning. Mice as in **a** were tested over two days, with performance assessed in 10 sessions, where fewer errors indicate improved memory and learning. Dara were analyzed via two-way RM ANOVA across all groups for day 2 and one-way ANOVA for block 10 with Bonferroni’s post-hoc, n = 19 (10M,9F) for APP V; n = 20 (10M,10F) for APP PU; n = 20 (11M,9F) for WT V; and n = 17 (9M,8F) for WT PU. **e** Percentage of freezing, representing fear response in individual mice as in **a**, measured before shock (baseline) and 24 hours later (contextual memory). Higher percentages of freezing indicate stronger associative memory of the aversive stimulus. Graph, mean ± s.e.m., one-way ANOVA with Bonferroni’s post-hoc, n = 17 (8M,9F) for APP V; n = 19 (10M,9F) for APP PU; n = 14 (8M,6F) for WT V; and n = 12 (6M,6F) for WT PU. See also Supplementary Fig. 10. Source data are provided as Source Data file.

Therefore, to test the hypothesis that epichaperomes directly contribute to memory dysfunction and validate our dfPPI findings, we assessed synaptic plasticity and memory through the 2-day radial-arm water maze (RAWM), fear conditioning, object location tasks (OLT), and LTP— a type of synaptic plasticity that is thought to underlie memory formation— in APP NL-F and WT mice treated with either an epichaperome disruptor, PU-AD, or with vehicle control (Fig. 9a, 10a). These tasks measure hippocampus-dependent functions such as spatial memory, associative learning, and recognition memory, which dfPPI indicated are dysregulated by epichaperomes. Each test reflects different aspects of the synaptic networks impacted by epichaperomes, providing direct functional readouts of the protein networks disrupted by epichaperome formation in AD.

For example, LTP directly reflects the ability of synapses to strengthen over time^80^. dfPPI analysis showed that epichaperome formation disrupted key protein networks involved in LTP, impairing synaptic strength and memory processes. OLT evaluates both short-term and long-term spatial memory, processes directly tied to synaptic plasticity and glutamatergic signaling^81^, which were disrupted in AD according to dfPPI analysis. Similarly, RAWM assesses short-term reference memory and spatial learning, functions that rely on intact hippocampal processes^82^. dfPPI identified networks involved in axon guidance and synaptic transmission as particularly affected by epichaperome formation, contributing to the spatial learning impairments observed in APP NL-F mice. Lastly, fear conditioning, which assesses hippocampal-amygdala interactions for associative memory^83^, also showed deficits related to epichaperome-mediated disruption.

**Figure 10.**
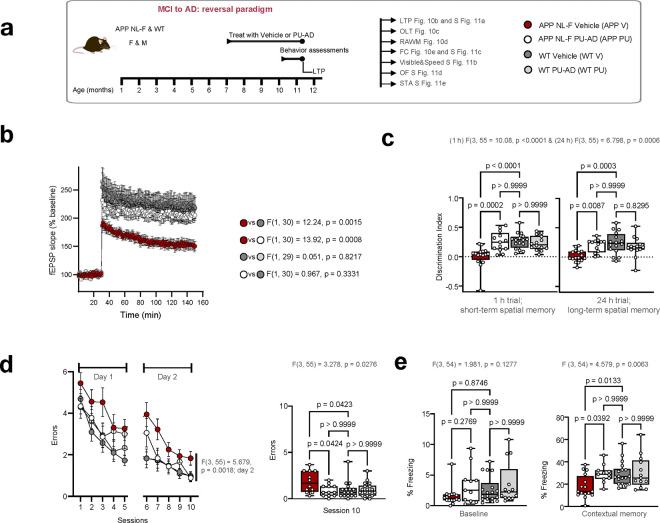
PU-AD treatment restores synaptic plasticity and memory deficits in APP NL-F mice. **a** Schematic of the experimental design for APP NL-F (APP) and WT littermates treated with vehicle (V) or PU-AD (PU) as indicated. Treatment started at an age where the mice already exhibit memory deficits (7 months of age), and continued for 3 months, until an age when the mice display severe impairments (10 months of age). OLT, object location task; RAWM, radial arm water maze; FC, fear conditioning; OF, open field; STA, sensory threshold assessment; LTP, long-term potentiation. **b** LTP measured as fEPSP slope (% baseline) over time in hippocampal slices from individual mice as in **a**. Graph, mean ± s.e.m., two-way repeated measures (RM) ANOVA. n = 16 (4M,5F) for APP V; n = 16 (4M,3F) for APP PU; n = 16 (5M,4F) for WT V; and n = 15 (4M,4F) for WT PU. **c** OLT data for mice as in **a** with short-term spatial memory determined at 1 h between the learning and the test trial, and long-term spatial memory with 24 h separation between the two trials. Mean ± s.e.m., one-way ANOVA with Bonferroni’s post-hoc, n = 16 (9M,7F) for APP V; n = 15 (7M,6F) for APP PU; n = 16 (7M,9F) for WT V; n = 14 (7M,7F) for WT PU. **d** RAWM performance: mean ± s.e.m. errors over sessions, analyzed via two-way RM ANOVA across all groups for day 2 and one-way ANOVA for block 10 with Bonferroni’s post-hoc, n = 12 (6M,6F) for APP V; n = 12 (7M,5F) for APP PU; n = 17 (8M,9F) for WT V; and n = 18 (9M,9F) for WT PU. **e** Percentage of freezing, representing fear response in individual mice, measured before shock (baseline) and 24 h later (contextual memory). Mean ± s.e.m., one-way ANOVA with Bonferroni’s post-hoc, n = 16 (9M,7F) for APP V; n = 11 (6M,5F) for APP PU; n = 18 (10M,8F) for WT V; and n = 13 (6M,7F) for WT PU. See also Supplementary Fig. 11. Source data are provided as Source Data file.

APP NL-F mice at 7 months exhibited significant synaptic plasticity impairments (measured by LTP) and memory dysfunction (assessed through the four behavioral tests) compared to age-matched WT mice (Fig. 9b–e). Given this trajectory of cognitive decline, we examined whether pharmacologic epichaperome disruption using the agent PU-AD^20^ could prevent or reverse impairments by treating mice presymptomatically (4–7 months, prevention, Fig. 9a–e) or postsymptomatically (7–10 months, reversal, Fig. 10a–e).

PU-AD treatment, given at the target engaging dose of 75mg/kg administered 3xweek^20^, successfully prevented and reversed LTP impairments. In the prevention group, 3-month PU-AD treatment rescued synaptic plasticity deficits compared to vehicle-treated controls (p=0.0006) (Fig. 9b). In the reversal group, PU-AD significantly restored LTP after synaptic deficits had already manifested (p=0.0008) (Fig. 10b). This demonstrates that PU-AD can both prevent and restore synaptic plasticity, returning LTP levels to those seen in WT mice, highlighting its potential in addressing synaptic dysfunction across disease stages. Additionally, PU-AD did not affect basal synaptic transmission, as shown by the input-output relationship in both WT and APP NL-F mice (**Supplementary Fig. 10a,11a**), confirming that PU-AD specifically targets pathological disruptions caused by epichaperomes without disturbing normal synaptic functions.

Similarly, PU-AD treatment effectively rescued both short-term and long-term spatial memory deficits in the OLT (Fig. 9c,10c). Untreated APP NL-F mice spent similar time exploring both moved and non-moved objects, indicating impaired spatial memory, while PU-AD-treated mice in both prevention and reversal paradigms showed a significant preference for the moved object, mirroring WT behavior (p=0.0006 for short-term, p=0.0002 for long-term memory in the prevention group; p=0.0024 and p=0.0066, respectively, in the reversal group). This suggests that PU-AD can restore hippocampal-dependent spatial memory impaired by epichaperome formation.

Further, RAWM testing revealed that PU-AD treatment significantly reduced spatial memory errors in both paradigms (p=0.0295 in prevention; p=0.0318 in reversal), effectively reversing deficits to levels comparable to WT mice (Fig. 9d,10d). Fear conditioning also showed significant recovery in contextual memory, with PU-AD treatment preventing and restoring deficits (p=0.0016 in prevention, p=0.0294 in reversal) (Fig. 9e,10e). Control experiments, including open field, visible platform, and sensory threshold tests, confirmed that these improvements were due to cognitive recovery rather than differences in motor or sensory function (**Supplementary Fig. 10b-e,11b-e**).

Crucially, PU-AD did not alter memory or LTP in WT mice (Fig. 9,10), confirming that epichaperome formation is specifically associated with disease processes, and PU-AD does not interfere with normal brain function. This underscores the disease-selective nature of epichaperomes and the precision of PU-AD in targeting pathological changes without affecting healthy neuronal processes.

The improvements in spatial memory and LTP following PU-AD treatment in APP NL-F mice provide strong evidence confirming the direct role of epichaperomes in driving synaptic dysfunction and cognitive impairment across the AD continuum. By preventing and reversing the synaptic disruptions in both early and late stages of the disease, PU-AD validates the dfPPI findings, showing that epichaperomes directly contribute to the network dysfunctions identified in both human and mouse models of AD.

Importantly, these results underscore not only the therapeutic potential of epichaperome disruption but also confirm that epichaperomes are a key driver of the cognitive dysfunction observed across the AD spectrum. The ability of PU-AD to restore normal synaptic function, particularly in glutamatergic and broader synaptic networks, highlights the direct link between epichaperome formation and synaptic PPI network dysfunction. This reinforces the idea that targeting epichaperomes could be an effective strategy for both preventing and reversing AD-related cognitive decline.

## Discussion

Our study demonstrates that epichaperomes play a central and active role across all stages of the AD spectrum, from the earliest preclinical phases through to advanced cognitive decline. Rather than being byproducts of disease, epichaperomes drive the progressive collapse of neuronal networks by persistently rewiring PPI interactions. This network-level disruption contributes to brain dysfunction, particularly affecting synaptic function and cognition, long before clinical symptoms manifest. By revealing how epichaperomes reshape molecular networks, our findings address a critical gap in AD research: understanding the mechanistic basis of early synaptic failure and progressive neurodegeneration. This represents a paradigm shift: rather than focusing on protein aggregation or on individual proteins, our study underscores the broader concept of network-targeting therapy to address the interconnected dysfunctions underlying AD pathology. This perspective complements existing models of AD, which have traditionally centered on amyloid and tau pathology.

Importantly, our findings underscore the progressive nature of epichaperome-mediated dysfunction: the higher the epichaperome levels, the more pronounced the network disruption. Our analysis reveals a dynamic trajectory of network remodeling, starting with localized disruptions in glutamatergic neurons and expanding to impact broader neuronal networks and brain regions as the disease progresses. This dynamic remodeling highlights epichaperomes as active and evolving contributors to AD pathology, rather than static features of diseased cells. As these pathological scaffolding platforms increase and extend to additional brain cells and regions, they co-opt new PPI networks, further exacerbating neuronal dysfunction. This progressive co-opting indicates that epichaperomes do not merely affect individual proteins or pathways. Rather, epichaperomes continuously and aberrantly reshape entire cellular networks, making network-targeting approaches particularly relevant.

A key insight from this study is that epichaperome formation disrupts neuronal protein function without altering gene expression or protein abundance. This challenges the prevailing view that AD pathology is primarily driven by transcriptional or proteomic changes. Instead, we identify maladaptive PPI network rewiring via epichaperome sequestration as an early and central driver of cognitive dysfunction.

Our results also highlight the selective vulnerability of glutamatergic synapses to epichaperome formation. These synapses, which mediate excitatory signaling in the brain, are critical for cognitive processes such as learning and memory^84,85^. Because glutamatergic neurons rely on finely tuned synaptic mechanisms, they are particularly sensitive to disruptions in PPI networks. The sequestration of Synapsin 1 into epichaperome platforms results in its mislocalization, impairing synaptic function at the earliest disease stages. This could provide a molecular explanation for why synaptic failure precedes the emergence of amyloid plaques or tau tangles in AD.

As AD progresses, epichaperome-driven dysfunction spreads beyond glutamatergic neurons, impacting inhibitory circuits and neuromodulatory pathways. This mirrors the evolving complexity of the disease process, where early memory deficits give way to broader cognitive impairments, such as executive dysfunction and attentional decline^86–88^. This shift from localized synaptic dysfunction to widespread network disruption reinforces the importance of early intervention—once epichaperomes form, they not only drive synaptic failure but also establish a framework for escalating neuronal dysfunction^16,24^.

Our findings provide strong evidence that epichaperome disruption, through pharmacological intervention, offers a compelling therapeutic strategy. The recovery of synaptic function and cognitive performance in APP NL-F mice, even at advanced stages of dysfunction, suggests that targeting epichaperomes could reverse functional impairments across the AD spectrum. Targeting epichaperomes, therefore, offers a network-centric therapeutic approach, emphasizing the potential of reversing systemic, multi-pathway disruptions through a single intervention that could potentially halt or even reverse the complex network failures seen in AD.

Beyond its mechanistic and therapeutic implications, this study provides a comprehensive systems-level map of progressive protein network dysfunction across the AD continuum. By leveraging dfPPI analysis across human and murine brain specimens, validated in mouse models and cellular systems, we define a trajectory of network disruption that begins with early miswiring and expands to broad dysfunctions in disease-associated pathways. This framework extends beyond AD, as PPI network dysregulation is a hallmark of many neurodegenerative and complex diseases, underscoring the broader applicability of network-based approaches in disease research. Additionally, the interactomics datasets generated here provide a valuable resource for the research community, enabling further investigations into the evolving architecture of disease-associated protein networks and their therapeutic potential. Together, these findings establish network biology as a critical framework for understanding disease progression and identifying novel intervention strategies.

In summary, this study redefines AD as a disease of progressive network instability, where the early miswiring of PPI networks—not merely protein aggregation—drives synaptic dysfunction and cognitive decline. We show that epichaperomes act as scaffolding platforms that orchestrate this dysfunction, revealing network dysregulation as both a disease driver and a therapeutic target. Crucially, we demonstrate that these pathological network changes are not only detectable and mappable but also reversible, opening new avenues for therapeutic intervention aimed at restoring network resilience. Moving forward, targeting epichaperomes or their downstream effects may offer a paradigm shift in AD treatment—one that prioritizes stabilizing disrupted protein networks rather than focusing on traditional disease hallmarks or single-protein targeting.

## METHODS

### Human biospecimens research ethical regulation statement

This research complies with all relevant ethical regulations for research involving human participants. Postmortem BA9 brain specimens from NCI, MCI, and AD cases were obtained from participants enrolled in the Rush Religious Orders Study (RROS)^27,89,90^. Each participant underwent comprehensive annual premortem clinical assessments and postmortem neuropathological evaluations. Exclusion criteria included evidence of mixed dementias, Parkinson’s disease, Lewy body disease, frontotemporal dementia, argyrophilic grain disease, vascular dementia, hippocampal sclerosis, and/or strokes/lacunes, as determined by a neurologist^23,80,81^. The RROS cohort offers a high-quality biospecimen tissue repository with low PMI case materials, high RNA integrity values, and optimal protein concentration, ensuring robust sample quality. A second cohort of BA9 brain tissue samples – designated as the NYUGSOM/NKI cohort - was obtained from tissue banks including the NIH Neurobiobank University of Miami Brain Endowment Bank (UMBEB), Center for Neurodegenerative Disease Research (CNDR), University of Pennsylvania School of Medicine, and the Johns Hopkins Medicine Institutional Brain Resource Center (JHMIBRC). Tissue integrity was confirmed for each case prior to conducting epichaperomics and associated biochemical and functional validation studies. Detailed data on age, sex, PMI, and additional variables are available in **Supplementary Data 1**. All tissue samples were collected with written informed consent and were de-identified before use in this research. While this study primarily focused on elucidating molecular mechanisms disrupted by epichaperome formation in AD, irrespective of sex, we aimed for a balanced distribution across sexes within each patient group to the extent possible. The distribution reflects a higher number of female participants, aligning with AD demographics. Accordingly, selection was not biased by sex, and sample composition reflects the natural prevalence of AD across sexes.

### Animal research ethical regulation statement

All animal studies were conducted in compliance with institutional guidelines and under the following Institutional Animal Care and Use Committee (IACUC) approved protocols: #05–11-024 and #04–03-009 for Memorial Sloan Kettering Cancer Center (MSKCC), AC-AABL0550 and AC-AABQ5559 for Columbia University, and #5832.8 and #6348.11 as per the Animal Resource Centre (ARC) administrative system at the University of Toronto. Founder male and female C57BL/6 mice (RRID:IMSR JAX:000664) were obtained from Jackson Laboratory (Bar Harbor, ME). Founder *APP*^*NL-F/NL-F*^ mice (RRID:IMSR_RBRC06343), referred to here as APP NL-F, were obtained from RIKEN BioResource Center (Tokyo, Japan). These mice are genetically engineered to carry humanized mutations in the amyloid precursor protein (APP) gene, specifically the Swedish (NL) and Iberian (F) mutations. These mutations increase the production of Aβ42 peptides and elevate the Aβ42 to Aβ40 ratio, closely mirroring the amyloid profiles seen in human AD^59^. Breeding colonies for each genotype were established using founder mice, and progeny from these colonies were used for all experiments. Genotypes were confirmed using a combination of primers (5’-ATCTCGGAAGTGAAGATG-3’; 5’-ATCTCGGAAGTGAATCTA-3’; 5’-TGTAGATGAGAACTTAAC-3’; 5’-CGTATAATGTATGCTATACGAAG-3’)^59^. The PCR template involves 35 cycles consisting of 94°C for 30 sec followed by 58°C for an additional 30 sec and 72°C for 30 sec and the reaction is quenched at 4°C. This protocol results in a wild-type band at 700 bp and the APP NL-F/NL-F positive mice at 400 bp. It should be noted that a non-specific band is occasionally observed slightly higher than the wild-type band and a positive sample should be run on the same gel to unequivocally identify the true wild-type animals. Mice were housed in groups of 4–5 per individually ventilated cage under a 12-hour light/dark cycle (lights on at 6:00 a.m. and off at 6:00 p.m.), with controlled temperature (22 ± 1 °C) and humidity (30–70%). Food and water were provided ad libitum. All mice were monitored daily for clinical signs to ensure animal welfare.

### Reagents and chemical synthesis

All commercial chemicals and solvents were purchased from Sigma Aldrich or Fisher Scientific and used without further purification. The PU-TCO epichaperome probe and the PU-NTCO control probe, as well as the dfPPI chemical probes - PU-beads and control beads - were generated using published protocols^23,26,32^. The identity and purity of each probe was characterized by MS, HPLC, TLC, and NMR. Purity of target compounds has been determined to be >95% by LC/MS on a Waters Autopurification system with PDA, MicroMass ZQ and ELSD detector and a reversed phase column (Waters X-Bridge C18, 4.6 × 150 mm, 5 μm) eluted with water/acetonitrile gradients, containing 0.1% TFA. Stock solutions of all compounds were prepared in molecular biology grade DMSO (Sigma Aldrich) at 1,000× concentrations.

### Cell lines and culture conditions

Differentiated iPSC-derived glutamatergic neurons (iGluts) were obtained from Fujifilm Cellular Dynamics (iCell GlutaNeurons, 01279, Catalog # R1116). Neurons were cultured according to the manufacturer’s instructions in a humidified, sterile environment at 37°C and 5% CO_2_. Before plating, 4-well glass plates (Cellvis, C4–1.5H-N) were coated with 15 mg mL^−1^ Poly-L-Ornithine (Sigma Aldrich, P3655) as a base layer, incubated 3 h at room temperature. Plates were then washed three times with HBSS and further coated with 0.028 mg mL^−1^ Matrigel solution (Corning, 354230), incubated overnight at 37°C. Neurons were seeded at 12.5 × 10^4^ cells per well. The neuron culture medium consisted of BrainPhys Neuron Medium (STEMCELL Technologies, 05790), iCell Neural Supplement B, iCell Nervous System Supplement, N-2 Supplement (Thermo Fisher Scientific, 17502–048), laminin (Sigma-Aldrich, L2020), and penicillin-streptomycin (Thermo Fisher Scientific, 15140–122), and was refreshed every two days. N2a cells (CCL-131, RRID: CVCL_0470) were purchased from ATCC and are derived from the brain tissue of a female mouse. PCR analysis confirmed the absence of male-specific Sry gene products. N2a cells were maintained in a humidified incubator at 37°C with 5% CO_2_ in DMEM (Thermo Fisher Scientific, 11965–092) supplemented with 10% FBS (Thermo Fisher Scientific, 10082–147) and 1% penicillin-streptomycin (Thermo Fisher Scientific, 15140–122). Culture media was changed every 2–3 days. All cell lines used in this study were tested and authenticated to be free of human infectious agents and mycoplasma contamination. They were verified through short tandem repeat profiling and underwent karyotyping to confirm the absence of significant chromosomal rearrangements post-reprogramming or differentiation. Cell line selection was not based on gender, sex, or ethnicity.

### Stressor preparation and cell treatment

Aβ42 oligomer (oAβ42), monomer and scramble were prepared as reported^91–93^. Briefly, lyophilized Aβ42 peptide (Anaspec Inc., AS-20276, 1 mg) was first dissolved in 100% 1,1,1,3,3,3-hexafluoro-2-propanol (HFIP) (Sigma-Aldrich, 105228–5G) to form a 1mM solution. HFIP disrupts any pre-existing aggregates, allowing the peptide to form a uniform monomeric solution. When the HFIP evaporates, it leaves behind a dried peptide film that is free of any initial aggregates or oligomers. The solution was allowed to evaporate, leaving a clear peptide film, which was subsequently stored at −20°C. The peptide film was resuspended in dimethyl sulfoxide (DMSO, Sigma-Aldrich) and sonicated for 10 min. This Aβ42-DMSO solution was then diluted in sterile PBS, vortexed for 30 s, and incubated at 4°C for 24 h to promote oligomer formation. The biochemical profile of the aged Aβ42 solution was characterized by using native PAGE and immunoblotting with anti-human Aβ monoclonal antibody 6E10 (BioLegend, 803001, RRID:AB_2564653, 1:1,000). Scrambled Aβ42 peptide (SCR) was purchased from AnaSpec Inc (AS-25382) and prepared as described above for oAβ42. Concentrations of Aβ42 were calculated based on the monomeric peptide’s molecular weight. For iGluts, on day 17 after plating, oAβ42 or SCR (100 nM) were added to the neuron cultures and plates were incubated for 24 h at 37°C and 5% CO_2_. For rescue studies, iGlut neurons (day 16 in culture) were first treated with 100 nM oligomeric Aβ42 (oAβ42) at 37°C for 24 hours to induce cellular stress and epichaperome formation. Subsequently, neurons were incubated with 1 μM PU-H71 at 37°C for various time points (2, 4, 8, 12, and 24 h) to assess the effects of epichaperome disruption. The effect of epichaperome disruption on Synapsin 1 localization was assessed using Synapsin 1 (D12G5) antibody (Cell Signaling Technology, 5297, RRID:AB_2616578, 1:200), with βIII Tubulin (2G10) (Abcam, ab78078, RRID:AB_2256751, 1:500) serving as a neuronal marker. For N2a neuronal cells, 2 × 10^6^ cells were seeded in a 60 mm cell culture dish. The following day, cells were treated with either DMSO (control) or the indicated concentrations of Aβ42 monomers or oligomers. After 24 hours, cells were harvested in 1× native lysis buffer for analysis by native PAGE, SDS-PAGE, and immunoblotting or incubated with PU-TCO for confocal microscopy analysis. For cell lysis and protein extraction, cells were collected in 20 mM Tris (pH 7.4), 20 mM KCl, 5 mM MgCl_2_, 0.01% NP-40 buffer with protease and phosphatase inhibitors (Roche) added. The samples were subjected to three rounds of freeze-thaw procedure to rupture the cells. The extracts were incubated at 4°C for 30 min and centrifuged at 13,000 × g for 10 min at 4°C. Supernatants were collected, protein concentrations were determined with BCA assay kit and samples were prepared for native PAGE and SDS-PAGE as described below.

### Mouse tissue collection

For tissue collection, animals were anesthetized by injection of sodium pentabarbital until unresponsive to toe pinch. Mice were perfused via transcardial puncture with ice-cold phosphate buffer using a 26-gauge needle attached to a perfusion pump. Perfusion was conducted for 2–3 min at 7.5 mL min^−1^ or until liver or the perfusate were clear. Brain tissue was carefully collected by opening the cranium and, immediately upon removal, brains were rapidly dissected, and samples were gently placed on pre-frozen (on dry ice) aluminum foil plate or metal plate. Extracted brains were covered with powdered dry ice to quickly freeze the tissue and avoid any distortion or post-mortem damage. Once completely frozen (1–2 min), tissues were stored in pre-chilled 5 mL tubes or scintillation vial and placed in a −80°C freezer until shipment on dry ice.

### Brain processing

For human brain samples, frozen tissue blocks from frontal cortex (BA9) were dissected from coronal tissue slabs^94^. Frozen tissue (approximately 50 mm^3^ in size) was homogenized on ice using a biomasher micro tissue homogenizer (K7496250010, Fisher Scientific) in a native lysis buffer containing 20 mM Tris (pH 7.4), 20 mM KCl, 5 mM MgCl_2_, 0.01% NP-40, along with protease and phosphatase inhibitors (Roche). The homogenates were incubated at 4°C for 30 minutes, then centrifuged at 13,000 × g for 10 min at 4°C to remove debris. Supernatants were collected, and protein concentrations were measured using a BCA assay kit (23225, Thermo Fisher Scientific) according to the manufacturer’s instructions. Protein levels were normalized based on HSP90α levels, and samples were processed for native PAGE, dfPPI, and SDS-PAGE as described below. For murine samples, frozen cortex tissue from APP NL-F and wild-type mice was similarly homogenized on ice using a micro tissue homogenizer (K7496250010, Fisher Scientific) in the same native lysis buffer used for human samples. Lysates were incubated at 4°C for 30 min and centrifuged at 13,000 × g for 10 min at 4°C. Supernatants were collected, protein concentrations were determined with a BCA assay kit (23225, Thermo Fisher Scientific), and samples were prepared for native PAGE, in-gel fluorescence, epichaperomics, and SDS-PAGE as outlined below.

### Native gel electrophoresis and western blot

Human Brain Samples. Epichaperome detection in postmortem frontal cortex samples was performed by loading normalized protein lysate aliquots onto 4–8% or 4–10% native gradient gels. Gels were resolved in Tris-Glycine buffer (25 mM Tris, 192 mM glycine) at 125 V for 2 h at 4°C. Following electrophoresis, proteins were transferred onto PVDF or nitrocellulose membrane in a buffer containing 0.02% SDS (25 mM Tris, 192 mM glycine, 20% (v/v) methanol) at 100 V for 2 h at 4°C. Membranes were blocked and then probed with primary antibodies against HSC70 (Enzo, SPA-815; RRID: AB_10617277; 1:2,000), HOP (Cell Signaling, 5670; RRID: AB_10828378; 1:2,000), and CDC37 (Cell Signaling, 4793; RRID: AB_10695539; 1:1,000) in 5% milk/TBS/0.1% Tween 20 at 4°C overnight. Blots were then washed and incubated with HRP-conjugated secondary antibodies: goat anti-rabbit (Southern Biotech, 4010–05; RRID: AB_2632593; 1:5,000) and goat anti-rat (3030–05; RRID: AB_2716837; 1:5,000). Enhanced Chemiluminescence Detection (ECL) reagent (Thermo Scientific, 32209) was used to visualize antibody signal. NativeMark protein standard (Invitrogen, LC0725) was included to determine protein complex molecular weights. Mouse Cortex Samples. To detect epichaperomes in mouse cortex by immunoblotting, 130 μg of total protein extract was loaded onto 4–8% native gradient gels, which were run at 125 V for 2 h at 4°C. After transfer, membranes were immunoblotted with HSC70 (Enzo, SPA-815; RRID: AB_10617277; 1:2,000) and HOP (Cell Signaling, 5670; RRID: AB_10828378; 1:2,000) primary antibodies. Secondary antibody incubation and signal detection were as described above for human samples. To detect epichaperomes via in-gel fluorescence, 130 μg of total protein extract was incubated with 1 μM PU-TCO in native lysis buffer on ice for 2 h. Samples were then resolved on 4–12% native gradient gels (Thermo Fisher Scientific, XP04120BOX) at 125 V for 2 h at 4°C. Following electrophoresis, gels were incubated with 700 nM Cy5-tetrazine in PBS for 15 min at room temperature to complete the click reaction. Gels were washed three times in PBS and imaged using the ChemiDoc MP system (Bio-Rad). The Alexa 546 channel (520–545 nm excitation, 577–613 nm filter) was used for native gel ladder visualization (Invitrogen, LC0725), and the Cy5 channel (650–675 nm excitation, 700–730 nm filter) was used to capture the cy5-clicked PU-TCO signal. Channels were merged in Image Lab 6.1 (Bio-Rad) to align bands with the molecular weight ladder. Alternatively, clicking was done in lysate rather than in gel. In this case, 130 μg of total protein extract was incubated with 1 μM PU-TCO in native lysis buffer on ice for 2 h. Then, 1 μM Cy5-tetrazine was added to the samples for click reaction and incubated for 10 min. Samples were then resolved on 4–12% native gradient gels (Thermo Fisher Scientific, XP04120BOX) at 125 V for 2 h at 4°C. Gels were imaged using the ChemiDoc MP system (Bio-Rad). The Alexa 546 channel (520–545 nm excitation, 577–613 nm filter) was used for native gel ladder visualization (Invitrogen, LC0725), and the Cy5 channel (650–675 nm excitation, 700–730 nm filter) was used to capture the Cy5-clicked PU-TCO signal. Channels were merged in Image Lab 6.1 (Bio-Rad) to align bands with the molecular weight ladder. In order to quantify epichaperome levels, a normalized aliquot of internal standard (normal, WT brain) was loaded onto each gel. Gels (whole lane signal) were quantified using UN-SCAN-IT software version 7.1 (Silk Scientific, Inc., Provo, Utah), and data were graphed and analyzed using GraphPad Prism 10 software (GraphPad, San Diego, California). Cell line protein extracts. To detect epichaperome levels in Aβ42 treated N2a cells, 50 μg total protein aliquots were loaded onto 4–10% native gradient gel and resolved in Tris-Glycine buffer as described above. After the electrophoresis, proteins were transferred to PVDF membrane in 25 mM Tris, 192 mM glycine, 20% (v/v) methanol buffer at 100V at 4°C. Membranes were then blocked in 5% BSA in TBS/0.1% Tween 20 for 1 h. The blots were then probed with an HSP90α antibody (ab2928; RRID: AB_303423; 1:6,000) from Abcam. The antibody signal was visualized as described above.

### Total chaperone quantification

For chaperone quantification, 10–30 μg of total protein lysate prepared in the native lysis buffer as described above, was subjected to SDS–PAGE and transferred onto PVDF or nitrocellulose membrane in the transfer buffer (25 mM Tris, 192 mM glycine, 20% methanol) at 100V for 1 h at 4°C. The membranes were then incubated in 5% milk/TBS/0.1% Tween 20 at 4°C overnight with the indicated antibodies: HSP90β (SMC-107; RRID:AB_854214; 1:2,000) from Stressmarq; HSC70 (SPA-815; RRID:AB_10617277; 1:1,000), and HOP (SRA-1500; RRID:AB_10618972; 1:1,000) from Enzo; HSP90α (ab2928; RRID: AB_303423; 1:6,000), from Abcam; HOP (5670; RRID:AB_10828378; 1:1,000) and GAPDH (2118; RRID: AB_561053, 1:10,000) from Cell Signaling Technologies, β-actin (A1978, RRID: AB_476692, 1:3,000) from Sigma-Aldrich. The blots were washed with TBS/0.1% Tween 20 and incubated with corresponding HRP-conjugated secondary antibodies: goat anti-mouse (1030–05, RRID: AB_2619742, 1:5,000), goat anti-rabbit (4010–05, RRID: AB_2632593, 1:5,000) and goat anti-rat (3030–05, RRID: AB_2716837, 1:5,000) (Southern Biotech). The chemiluminescent signal was detected with ECL reagent (32209, Thermo Scientific) according to manufacturer’s instructions. Thermo Scientific PageRuler Plus prestained protein ladder (Fisher Scientific, 26619) or Precision Plus protein standards (Bio-Rad, 161–0375) were used as size standards in protein electrophoresis and Western blotting.

### Covariate assessment for the human cohort

Covariate data, including Braak stage, MMSE scores, amyloid density, and tangle density, were accessed from the Rush Alzheimer’s Disease Center Research Resource Sharing Hub (https://www.radc.rush.edu/docs/var/standardDatasetVariables.htm). The RADC assessed these variables using established neuropathological and cognitive evaluation methods, as described below, and provided them for correlative analyses in our study comparing epichaperome-high and -low groups. Briefly, Braak staging was conducted as a semiquantitative measure of neurofibrillary tangle (NFT) pathology based on Bielschowsky silver staining across key brain regions, including the frontal, temporal, parietal, entorhinal cortex, and hippocampus. NFTs were visualized and staged according to their distribution and severity as follows: Braak stages I and II (NFTs mainly confined to the entorhinal cortex), stages III and IV (limbic region involvement, such as the hippocampus), and stages V and VI (moderate to severe neocortical NFT involvement). Braak stages were assigned in alignment with established neuropathological criteria, informed by both an algorithmic approach and expert neuropathologist assessment^30^. The cognitive function of participants was assessed through the MMSE or estimated MMSE score when MMSE was unavailable. The MMSE, scored from 0 to 30, is a standardized 30-item dementia severity screening tool that evaluates orientation, recall, memory, arithmetic, and language skills, among other cognitive domains. For individuals unable to complete specific items, the score was adjusted as the sum of completed items, scaled to a 30-point equivalent^95^. Amyloid density was calculated as the mean square-root-transformed value of amyloid staining across eight brain regions: angular gyrus, anterior cingulate, calcarine cortex, entorhinal cortex, hippocampus, inferior temporal cortex, midfrontal gyrus, and superior frontal cortex. Immunohistochemistry was performed using 3 monoclonal antibodies against Aβ, 4G8 (1:9,000; Covance Labs, Madison, WI), 6F/3D (1:50; Dako North America, Inc., Carpinteria, CA), and 10D5 (1:600; Elan Pharmaceuticals, San Francisco, CA). Tangle density quantification similarly covered the same eight brain regions, using a square-root-transformed mean value for regional tau-tangle density. Immunohistochemistry was performed using an antibody specific to phosphorylated tau, AT8 (1:2000, ThermoScientific). Values were averaged across regions when at least four of eight regions were available^31^.

### Epichaperome staining in N2a cells

To quantify epichaperome levels, N2a cells treated with Aβ42 were incubated with 1 μM PU-TCO for 1 hour at 37°C in a 5% CO_2_ incubator. Following incubation, cells were washed three times with 1× PBS and fixed with 4% formalin for 15 minutes. Fixed cells were then washed three additional times with 1× PBS and permeabilized with 0.1% Triton X-100 in 1× PBS for 15 minutes. After permeabilization, cells were washed three times with 1× PBS. Click labeling was performed by incubating the cells with 700 nM Cy5-tetrazine (Cat. No. 1189–1, Click Chemistry Tools) in 1× PBS for 15 minutes. Excess reagent was removed by washing the cells three times with 1× PBS to reduce background fluorescence. Coverslips were mounted with ProLong^™^ Gold Antifade Mountant with DAPI, and imaging was performed using a Zeiss LSM880 point-scanning confocal microscope equipped with AiryScan technology. Images were analyzed using ImageJ (v1.54f, Java 1.8.0_322, 64-bit). For quantification, TIFF files were opened in ImageJ, and the scale was set according to the metadata. Regions of interest (ROIs) were selected and added to the ROI Manager tool. For each ROI, the area (in μm^2^) and mean fluorescence intensity (MFI) were measured. The MFI per μm^2^ was calculated and plotted, and data were subjected to statistical analysis using GraphPad Prism 9.

### Epichaperome staining in iCellGluta neurons

To visualize epichaperomes, glutamatergic neurons were incubated with 1 μM PU-TCO (epichaperome probe) and 1 μM PU-NTCO (control inert probe) for 1 h at 37°C. Epichaperome staining procedure was performed as previously described^32^. Briefly, neurons were fixed in 4% paraformaldehyde (PFA, Thermo Fisher Scientific, 043368.9M) at room temperature (RT) for 15 min, permeabilized with 0.2% Triton X-100 (MP Biomedicals, 194854) in PBS for 15 min at RT. The click reaction to attach Cy5-tetrazine to PU-TCO was performed by adding 700 nM Cy5-tetrazine (Click Chemistry Tools, 1189–1) in PBS for 12 min at RT. The samples were blocked with 1% Bovine Serum Albumin (BSA) diluted in PBS for 30 min at RT. The neurons were incubated overnight at 4 °C in blocking solution containing the primary antibodies against beta III Tubulin (2G10) (Abcam, ab78078, RRID:AB_2256751, 1:500), Synapsin-1 (D12G5) (Cell Signaling Technology, 5297, RRID:AB_2616578, 1:200) and PSD95 (Abcam, ab12093, RRID:AB_298846, 1:100). After washing with PBS, the following secondary antibodies were used: Alexa Fluor^™^ 488 Goat anti-Mouse IgG (H+L) Cross-Adsorbed Secondary Antibody (Thermo Fisher Scientific, A-11001, RRID:AB_2534069, 1:500), Alexa Fluor^™^ 568 Goat anti-Rabbit IgG (H+L) Cross-Adsorbed Secondary Antibody (Molecular Probes, A-11011, RRID:AB_143157) and Alexa Fluor^™^ 568 Donkey anti-Goat IgG (H+L) Cross-Adsorbed Secondary Antibody (Thermo Fisher Scientific, A-11057, RRID:AB_2534104). The nuclei were counterstained with 200 nM Hoechst 33342 (Thermo Fisher Scientific, 62249) in PBS for 5 min. Imaging was performed using Zeiss LSM880 with Airyscan (Zeiss Inc., Oberkochen, Germany) under 63× oil-immersion Zeiss plan-apochromat 63×/NA 1.4 objectives. Mean fluorescence intensity (MFI) measurements were performed for whole neurons and subcellular compartments (perinucleus, nucleus, and projections) across experimental groups, including the control group, scrambled Aβ42 treatment group, and oAβ42 treatment group. For rescue experiments with PU-H71, perinuclear Synapsin 1 intensities were measured for all experimental groups: no treatment (control), oAβ42 treatment, 2-hour PU-H71 treatment, 4-hour PU-H71 treatment, 8-hour PU-H71 treatment, 12-hour PU-H71 treatment, and 24-hour PU-H71 treatment. Additionally, Synapsin 1 cluster intensities were manually measured from randomly selected clusters (five clusters per cell) in both control neurons (n = 100 clusters) and oAβ42-treated neurons (n = 100 clusters). All fluorescence intensity measurements were performed using Zen Blue software (Zeiss Inc., Version 3.7), ensuring consistent analysis across experimental conditions. 3D rendering and visualization were performed using Imaris software (Oxford Instruments, version 9.5.1). Animations were created 1600 × 1200 pixels with 1200 frames both vertical and horizontal dimensions. Thresholds were set according to control groups (PU-NTCO for PU-TCO and secondary antibody control and single labeling for multiple labeled cells). To assess the restoration of projection morphology, we manually measured the thickness of projections (in nanometers) using ImageJ (Fiji, Version 1.53t). After setting the scale, the straight-line tool was used to measure the diameters of the projections. Measurements were taken from three different regions for every projection and averaged.

### Colocalization analysis

All the data collection procedures for the variables that could impact the imaging outcomes—such as cover glass, filters, gain settings, and laser power—were consistent throughout the study. Colocalization analysis was performed using ImageJ (Fiji, Version 1.53t). We implemented multiple control groups, which included secondary antibody staining, single-label control, and negative staining. The same microscope settings used for the double-label studies were also applied when capturing images of these controls. For samples with two labels, we applied the same pixel distribution threshold as for single labels. Co-localized images were created using the “AND” operator in the Image Calculator after thresholding and segmenting the dual-labeled cells. Once the images were captured, pixels in various cellular compartments (perinucleus, nucleus and projections) were counted. Using the total number of epichaperome-positive pixels and the marker (i.e., Synapsin 1 or PSD95)-positive pixels, we calculated the percentage of epichaperomes associated with the markers and the percentage of markers found in the epichaperome for all the markers analyzed.

### Epichaperome staining in mice

APP NLF and wild type-mice were sacrificed at 3, 7, or 14 months of age. Mice were anesthetized with ketamine (83 mg kg^−1^) and xylazine (13 mg kg^−1^) in 0.9% sterile saline and transcardially perfused with ice-cold PBS. Whole brains were removed, snap-frozen on dry ice, and stored at −80°C until staining^32^. Briefly, brains were embedded in OCT (Tissue-Tek, Sakura), frozen, and sectioned into 20 μm coronal or sagittal slices using a cryostat (Leica, VT100S). Epichaperome staining was conducted by incubating cryosections with 1 μM PU-TCO (epichaperome probe) or 1 μM PU-NTCO (control probe) diluted in SuperBlock Blocking Buffer (SBB) (Thermo Fisher Scientific, 37515). Sections were washed twice with PBS for 15–30 s, and then were fixed with 4% PFA in PBS for 30 min at RT and subsequently rinsed two times with PBS for 15–30 s. Following fixation, sections were permeabilized with 0.02% Triton-X100 in PBS for 15 min and then sections were rinsed two times in PBS. To perform the click reaction, brain sections were incubated with freshly prepared Cy5 Tetrazine (700 nM, diluted in SuperBlock Blocking Buffer) for 15 min at RT. After washing twice with PBS, brain slices were blocked with blocking solution (0.3% Triton-X100 in SuperBlock Blocking Buffer) for 1 h at RT. The sections were then placed in blocking buffer with Anti-NeuN [1B7] antibody (Abcam, ab104224, RRID: AB_10711040, 1:500), Anti-MAP2 antibody (Abcam, ab32454, RRID:AB_776174, 1:250), and Anti-Glial Acidic Fibrillary Protein (GFAP) antibody (Abcam, ab53554, RRID:AB_880202, 1:250) overnight at 4°C. After two PBS rinses, sections were incubated with species-specific secondary antibodies (1:500) for 3 hours at room temperature, and Hoechst (ThermoFisher Scientific, 62249, 1:1000 dilution in PBS) was used as a nuclear counterstain. Sections were mounted with Glycerol/PBS. Slides were scanned with a Pannoramic Scanner (3DHistech, Budapest, Hungary) using a 20×/0.8 NA objective, and intensity measurements of brain regions were performed with ImageJ/FIJI software (Version 1.54f). For super-resolution and confocal imaging, sections were visualized using a Zeiss LSM880 with Airyscan and a 40× oil immersion objective.

### Sample preparation for dysfunctional Protein-Protein Interactome (dfPPI) analysis

PU-beads and control beads were washed three times with 20 mM Tris (pH 7.4), 20 mM KCl, 5 mM MgCl_2_, and 0.01% NP40 (native lysis buffer) before use. For human brain extracts, 20 μL of control beads were distributed into Eppendorf tubes, and normalized protein extracts were added in a final volume of 250 μL with native lysis buffer. Samples were incubated at 4°C for 30 min with rotation and centrifuged at 10,000 × g for 1 min at 4°C to remove protein aggregates. Then, 40 μL of PU-beads were transferred into new Eppendorf tubes, and the supernatant from the control beads was added. Samples were incubated with rotation at 4°C for 3 h, followed by four washes with native gel buffer. After washing, 10 μL of 5× Laemmli buffer was added to the beads, and samples were heated to 95°C for 3 min. After centrifuging at 10,000 × g for 1 min, the supernatant was collected for SDS-PAGE. After the run, gels were stained with Coomassie G-250 (Bio-Rad, 1610786), and further processed for LC-MS. For mouse brain samples, 20 μL of control beads were prepared in Eppendorf tubes, and 250 μg of total protein extract was added in a final volume of 250 μL with native lysis buffer. Samples were incubated for 30 min at 4°C, centrifuged at 10,000 × g for 1 min, and the supernatant was transferred to 40 μL PU-beads. After a 3 h incubation at 4°C with rotation, beads were washed three times with 20 mM Tris (pH 7.4), 20 mM KCl, 5 mM MgCl_2_, and 0.01% NP40 buffer, followed by two washes with PBS. The samples were then submitted for on-bead protein digestion and LC-MS.

### Protein identification

For the mouse brains, following interactome capture on PU-beads, the cargo was subjected to on beads protein digestion at the Proteomics Core of MSKCC. The beads were resuspended in 80 μL of 2 M Urea, 50 mM Ammonium Bicarbonate (pH 8.5) and treated with DL-dithiothreitol (1 mM final concentration) for 30 min at 37°C with shaking (1100 rpm) on a Thermomixer (Thermo Fisher). Free cysteine residues were alkylated with 2-iodoacetamide (3.67 mM final concentration) for 45 min at room temperature at 1100 rpm in the dark. The reaction was quenched by adding DTT (3.67 mM final concentration). LysC (750 ng) was added, followed by incubation for 1 h at 37°C at 1150 rpm before digestion with trypsin (750 ng) at 37°C at 1150 rpm overnight. After incubation, the peptide digest was acidified to pH <3 by adding 50% trifluoroacetic acid (TFA), and the peptides were desalted using 3-plug C18 stage tips (3M EmporeTM high performance extraction disks). The stage tips were conditioned sequentially with: i) 100 μL methanol, ii) 100 μL 70% acetonitrile (ACN)/0.1% TFA, iii) 100 μL 0.1% TFA, iv) 100 μL 0.1% TFA. After conditioning, the acidified peptide digest was loaded onto the stage tip. The stationary phase was washed with 100 μL of 0.1% formic acid (FA), and the peptides were eluted using 50 μL of 70% ACN/0.1% FA twice. Eluted peptides were dried under vacuum and reconstituted in 12 μL of 0.1% FA, followed by sonication and transfer to an autosampler vial. Peptide yield was quantified using a NanoDrop (Thermo Fisher). Peptides were separated on a 25 cm column, 75 μm diameter and 1.7 μm particle size of C18 stationary phase (IonOpticks Aurora 3 1801220), using a gradient from 2% to 95% Buffer B over 90 minutes (Buffer A: 0.1% FA in HPLC grade water; Buffer B: 99.9% ACN, 0.1% FA) at a flow rate of 300 nL/min using a NanoElute 2 system (Bruker). MS data were acquired on a TimsTOF HT (Bruker) with a Captive Spray source (Bruker) using a data-independent acquisition PASEF method (diaPASEF). The mass range was set from 100 to 1700 m/z, with an ion mobility range of 0.60 V.s/cm^2^ (collision energy: 20 eV) to 1.6 V.s/cm^2^ (collision energy: 59 eV), a ramp time of 100 ms, and an accumulation time of 100 ms. The dia-PASEF method used a mass range of 400.0 to 1201.0 Da, a mobility range of 0.60–1.60, and an estimated cycle time of 1.80 seconds. The dia-PASEF windows were set with a mass width of 26.00 Da, a mass overlap 1.00 Da, and 32 mass steps per cycle. Raw data files were processed using Spectronaut version 18.5 (Biognosys) and searched with the PULSAR search engine with a Mus musculus UniProt protein database downloaded on 2024/05/28 (96,014 entries). Cysteine carbamidomethylation was set as fixed modification, while methionine oxidation, protein N-terminus acetylation, and deamidation (NQ) were set as variable modifications. A maximum of two trypsin missed cleavages were permitted. Searches used a reversed sequence decoy strategy to control peptide false discovery rate (FDR), with a threshold of 1% FDR for identification. For the human brains, and after elution from PU-beads, proteins were purified and resolved by SDS-PAGE Gels were washed 3 times in double-distilled water for 15 min each and proteins visualized and fixed by staining with EZ-Run Protein staining solution (ThermoFisher Scientific, USA). Stained protein gel regions were cut into 5 gel sections and de-stained 3 times in 50% methanol for 15 min each time. Any residual stain was removed by 50 mM NH_4_HCO_3_ in 30% acetonitrile. Dried gel samples were digested overnight with mass spectrometry grade trypsin (Trypsin Gold, Promega, Madison, WI, USA) at 5 ng μL^−1^ in 50 mM NH_4_HCO_3_ digest buffer. After acidification with 10% formic acid, peptides were extracted with 5% formic acid/50% Acetonitrile (v/v) and concentrated to a small droplet using vacuum centrifugation. Desalting of peptides was done using hand packed SPE Empore C18 Extraction Disks as described^96^. Desalted peptides were again concentrated and reconstituted in 10 μL 0.1% formic acid in water. Aliquots of the peptides were analyzed by nano-liquid chromatography followed by tandem mass spectrometry (nano-LC-MS/MS) using an Easy nLC 1000 equipped with a self-packed 75 μm × 20 cm reverse phase column (ReproSil-Pur C18, 3 μm, Dr. Maisch GmbH, Germany) coupled online to a QExactive HF Orbitrap mass spectrometer via a Nanospray Flex source (all instruments from Thermo Fisher Scientific, Waltham, MA, USA). Analytical column temperature was maintained at 50°C by a column oven (Sonation GmBH, Germany). Peptides were eluted with a 3–40% acetonitrile gradient over 110 min at a flow rate of 250 nL min^−1^. The mass spectrometer was operated in data-independent acquisition mode^97^; MS survey scans were acquired in profile mode, at a resolution of 120,000 (at m/z 200) over a scan range of 300–1650 m/z. Following the survey scans, 30 groups of precursors were selected for fragmentation with sliding isolation windows to include peptide m/z values ranging from 364 to 1370 Th. The default maximum charge state was set to 4 and resolution was set to 30,000. In MS/MS, the fixed first mass was set to 200 Da. The normalized collision energy (NCE) /stepped NCE was 25.5,27,30. The maximum injection times for the survey scan was 60 ms and for MS/MS, it was set to auto. The ion target value for both scan modes was set to 3e6. Data were analyzed by the software MaxQuant (version 2.1.1.0), referred to as MaxDIA analysis type. To identify peptides, we used human in silico-generated spectral libraries that were created from the Uniprot human protein sequence database (downloaded on 06/18/2020; 20,956 entries). The library was obtained from the Max Plank Institute of Biochemistry Data share drive (MPIB Datashare https://datashare.biochem.mpg.de/s/qe1IqcKbz2j2Ruf?path=%2FDiscoveryLibraries). To determine the relative abundance of a protein across a set of samples, we used its label free quantitation (MaxLFQ) intensity calculated by MaxDIA^97^, which normalizes the protein intensity by normalizing peptide intensities across samples.

### Bioinformatics analyses

Mass spectrometry (MS) intensity values from proteins identified through differential protein-protein interaction (dfPPI) analysis were further processed for pathway enrichment using gProfiler^98^. For human samples, pairwise comparisons were conducted across all disease conditions, specifically AD vs. NCI, AD vs. MCI, and MCI vs. NCI. Proteins with a fold change (FC) greater than 1 and a t-test p-value of ≤ 0.25 in each comparison were subjected to gProfiler. This threshold was chosen based on prior demonstration of its effectiveness in differential protein-protein interaction (dfPPI) analyses, as previously described^21^, where it was shown to balance sensitivity and specificity for detecting significant protein interactions. gProfiler analyses were performed using the following parameters: version e111_eg58_p18_f463989d, Homo sapiens as the organism, and query length of 4197. For the mouse dfPPI data, MS intensity values were analyzed for pathway enrichment through pairwise comparisons of proteins from 7-month-old and 15-month-old samples. Proteins showing a FC greater than 1 with a t-test p-value of ≤ 0.25 in each comparison (7 months > 15 months and 15 months > 7 months) were selected for enrichment analysis using gProfiler. The analysis employed the following parameters: version e111_eg58_p18_f463989d, with Mus musculus specified as the organism and a query length of 2063. We selected annotated domains only and used the significance threshold method of false discovery rate (FDR) with a user threshold of 0.05. The sources queried included KEGG, Reactome (REAC), and WikiPathways (WP). The query parameters excluded non-experimental evidence annotations, non-annotated domains, underrepresented measures, and multiple queries, while enabling result highlighting. Detailed datasets and analyses for human and mouse specimens are provided in Supplementary Data 2 and Supplementary Data 5, respectively. Pathways showing either consistent upregulation or downregulation across AD stages were selected for further investigation, and the results were visualized in Cytoscape (v3.10.2)^99^ using the reported pathway map template^20^. Each node in the Cytoscape visualization represents a pathway comprising a collection of different proteins. Nodes are functionally interconnected in a hierarchical manner (high to low, indicated by arrowheads) and displayed as a pathway tree. The number of proteins from our experimental list intersecting with each pathway is reflected in the size of the node, while the significance of enrichment is indicated by the node’s color. Pathways without any related proteins were represented as borderless gray nodes.

### Functional and mechanistic validation in mice

#### Animal study design

Sample size determination followed standard practices in the field. Specifically, behavioral and electrophysiological study sizes were based on previously published studies^100^, which have established sufficient sample sizes to measure the expected effect sizes. Sample sizes for each experiment are detailed in the corresponding figure legends. For behavioral assessments, mice were included in the analysis unless they met predefined exclusion criteria: animals that froze above 15% during baseline prior to foot shock in the fear conditioning test or explored objects for less than 10 seconds in the object location test were excluded. All behavioral assays were conducted multiple times using independent mice across separate cohorts to ensure reproducibility. For electrophysiological studies, 1–4 slices were recorded per mouse, and the number of slices and animals is specified in the respective graphs. Mice were randomly allocated to experimental groups. While the order of behavioral tests was not randomized due to animal welfare considerations, age- and sex-matched mice were balanced across experimental groups within each batch based on genotype. All behavioral and electrophysiological assessments were conducted with the investigators blinded to group allocations. Manual quantifications were performed in a blinded manner, and where possible, analyses were conducted using automated software for objectivity. Exceptions to software-based analysis included the object location test, radial-arm water maze, and sensory threshold assessment, where manual analysis was required.

### Epichaperome disruptor treatment

The epichaperome disruptor drug candidate PU-AD×2HCl was formulated in 30% Captisol in 60 mM Citrate Buffer (pH = 4). Treatment with the target saturating dose of PU-AD (75 mg kg^−1^)^19,20^ or Vehicle control started at the age of 4 months for the prevention group and at the age of 7 months for the reversal group. PU-AD was injected intraperitoneally three times a week (Monday-Wednesday-Friday) for 3 months prior to the commencement of the behavioral studies and continued for the duration of behavioral studies (3–5 weeks). All mice in all studies were observed for clinical signs at least once daily.

### Object Location (OLT)

For the OLT, we utilized a circular arena (40 cm in diameter) in which the back half is made of grey polyvinyl chloride (PVC) and the front half is made of transparent PVC. The test was performed in a dark room with a red fluorescent bulb for illumination. We used 2 different sets of two identical objects, which were divided in a semi-random manner between animals. The objects consisted of (1) a massive metal rectangular prism (2.5 cm × 5 cm × 7.5 cm) containing two holes (diameter 1.5 cm) and (2) a massive aluminum cube with a tapering top (4.5 cm × 4.5 cm × 8.5 cm). Habituation of the animals to the arena, the experimenter and handling was conducted in two consecutive days in which mice were introduced to the arena for 10 min to explore the first or the second set of objects. During the third day of the test, we evaluated short-term spatial memory by imposing an inter-trial interval of 1 h between the learning and the test trial, while long-term spatial memory was assessed on the fourth and fifth day when an interval of 24 h separated the two trials. For both versions of the test, mice were placed in the arena containing one set of identical objects and allowed to explore them for 10 min. During the learning trial, both objects were placed in the middle of the arena having the same predetermined distance with each other, whereas during the test trial one of the objects of the learning trial was moved to a different position on a vertical line, closer to the wall of the arena, changing the configuration of the arena. Mice were left to explore the objects for 10 min, and their exploration time was scored manually in both trials. Exploratory behavior was considered as pointing the nose to the object in a close distance (no more than 2 cm) and/or touching the object with the nose. Sitting next to the object was not considered exploration. The discrimination index that represents preference towards exploring the moved object was calculated by dividing the exploration time between the two objects during the test trial by the total exploration of the two objects during this trial.

### Radial Arm Water Maze

The RAWM test was performed in a circular pool, 120 cm in diameter, that was made opaque with a non-toxic white paint. An apparatus consisting of six arms radiating from the center was placed within the pool, and spatial cues were present within the room. The platform (10 cm diameter) was placed at the end of one of the arms, submerged in the water. Although the location of the platform was kept constant for each mouse, the starting position differed between the trials. The mice were trained on finding the platform (submerged 1 cm beneath the water surface) for two consecutive days, with 15 trials taking place each day per mouse. For facilitating the learning process, during the first day of the test, the platform was alternating between visible (flagged with an orange bottle cup) or hidden for the first 12 trials. The last 3 trials of the first day and all 15 trials of the second day the platform was hidden. In each trial, the mouse was placed in the starting arm and was allowed to swim for 60 s or until finding the platform. If the mouse could not find the platform within 60 s, the experimenter was guiding it to the platform. Once on the platform, the mouse was allowed to rest for 20 s and observe the visual cues. During each trial, an error was registered if the mouse was entering any other arm than the goal arm or if the mouse was not making a decision within 10 s. An entry was considered when all four paws of the mouse were into the arm. For preventing over-practice or fatigue, the trials were spaced by dividing mice in cohorts, and alternating the cohorts over the course of the 15 trials each day. The performance of the animals was depicted as the number of errors in which each block represents the average errors of three consecutive trials.

### Visible platform testing

The test was performed in the same conditions as RAWM test for the assessment of visual-locomotor-motivational deficits. The performance of mice was recorded in two consecutive days in which two set of trials were taken place each day. In each trial, the platform was placed in one of the three quadrants of the pool. The starting position was kept constant for a specific location of the platform. The mouse was placed on the water facing the wall of the pool and was allowed to swim until finding the flagged platform or until 60 s were passed. If the mouse was not finding the platform within this time, it was guided to it by the experimenter. The mouse was allowed to stay in the platform for 20 s and observe environmental cues. The latency and velocity before reaching the platform were tracked by the Ethovision XT video system. The graphs are shown the results of 4 blocks with each block representing the average performance in one set of experiments.

### Fear conditioning

The fear conditioning test was employed for assessing associative memory and took place in three consecutive days. On the first day, mice were placed in the fear conditioning chamber (Noldus) for 2 min before the appearance of a tone (conditional stimulus; 2880 Hz at 85 Db). Mice receive a foot shock (unconditional stimulus 0.8 mA) during the last 2 s of the tone. After pairing of the 2 stimuli, mice were left in the chamber for another 30 s in the absence of a stimulus. After 24 h, mice were placed back to the fear conditioning chambers for 5 min without the presence of tone or shock. Freezing behavior was recorded with a vision tracking system (Ethovision XT, Noldus). The third day, the cued fear memory was assessed by placing the mouse in a modified chamber (different floor, walls and addition of vanilla odor) that represents a novel context. During the first 2 min of the 5-min trial, the mice were allowed to explore the novel chamber in the absence of any stimuli, while the last 3 min, they were exposed in the tone of the first trial. The freezing behavior before and during the conditional stimulus was tracked by the Ethovision system.

### Open field

The test was employed for evaluating exploratory behavior and anxiety levels in mice. During two consecutive days, each mouse was placed in the open field arena consisting of plexiglass transparent walls (model ENV- 520; Med Associates, St. Albans, Vermont, 43.2 cm long × 43.2 cm width × 30.5 cm high) and were allowed to explore it for 10 min. The time spent in the center and the total entries in the center were recorded by a vision tracking system.

### Sensory threshold assessment

The test was performed at the last day of the experiments for evaluating pain perception of the shock. The animals were placed in the fear conditioning chamber and received foot shock for 1 s of increasing intensity, ranging from 0.1 to 0.6 mA at 0.1 mA increments every 30 s. Mice were recorded by a vision tracking system (Ethovision XT) and responses to shock were evaluated. Specifically, the intensity of shock that elicited the first visible response (flinching), the second motor response (jumping), and the first audible response (vocalization) was recorded manually by the experimenter.

### Electrophysiological recordings

Mice were sacrificed through cervical dislocation and brains were excised immediately after decapitation. Hippocampi were removed and transverse hippocampal slices of 400 μm thickness were cut on a tissue chopper and transferred to the recording chamber to recover for at least 90 min. The conditions in the recording chamber resembled the physiological conditions in the brain. In more detail, the slices were continuously perfused with artificial cerebrospinal fluid (ACSF) bubbled with 95% O_2_ and 5% CO_2_, and temperature was maintained at 30±0.5°C. The ACSF composition was the following (mM): NaCl (124.0), KCl (4.4), Na_2_HPO_4_ (1.0), NaHCO_3_ (25.0), CaCl_2_ (2.0), MgCl_2_ (2.0), and glucose (10.0). Field extracellular post-synaptic potentials (fEPSP) were measured at the Schaeffer collateral of the hippocampus. For that, a bipolar tungsten electrode was placed at the Schaeffer collateral fibers and a glass electrode filled with ACSF was placed in the stratum radiatum of CA1. Basal synaptic transmission was evaluated by measuring the evoked fEPSP responses in increased stimulation voltages (5, 7, 9, 10, 11, 12, 13, 14, 15, 25, 30, 35 V), generating the Input-Output (I-O) curve. The baseline of the long-term potentiation (LTP) recordings was recorded at approximately 35% of the maximum evoked slope, as determined by the I-O curve. After 30 min of stable baseline, LTP was induced by theta-burst tetanization (four pulses at 100 Hz, with the bursts repeated at 5 Hz and each tetanus including three 10-burst trains separated by 15 s) and slopes were recorded for 2 h after stimulation. LTP was expressed as percentage of the baseline and results were analyzed in pClamp 11 (Molecular Devices).

### Statistics and reproducibility

Unless as specified above under Protein identification and Bioinformatics analyses, statistics were performed, and graphs were generated, using Prism 10 software (GraphPad). Most data are presented as either violin plots or box-and-whisker plots displaying minimum to maximum values, with individual data points representing all values in the dataset. Violin plots show the distribution and density of the data, with medians and quartiles indicated. For box-and-whisker plots, the interquartile range is boxed, and the median is marked within the box. Statistical significance was determined using ANOVA, with specifics indicated for each analysis. For electrophysiological recordings, fEPSP slopes of Input-Output and LTP were analyzed by two-way ANOVA for repeated measures. For the behavioral tests, animals were run in cohorts in which sex of mice was kept balanced across groups. Results were analyzed with ANOVA for repeated measures (RAWM errors, latency and speed) or one-way ANOVA with Bonferroni post-hoc correction (OLT, 10TH block of RAWM, FC, OF and STA). Differences were considered significant at a p value less than 0.05. Results were expressed as mean ± Standard Error of the Mean (SEM). No statistical methods were used to pre-determine sample sizes, but these are similar to those generally employed in the field. No samples were excluded from any analysis unless explicitly stated.

## Data Availability

The source data underlying all main and supplementary figures – raw data, statistical analyses and uncropped gels - are provided with this paper as a Source Data file and were deposited in the Figshare repository under accession code [in progress]. Datasets and analytics associated with dfPPI and quantitative proteomics analyses are available in the Supplementary Information as Supplementary Data 1 through 6 and were deposited in the Figshare repository under accession code [in progress]. LC-MS data, including dfPPI raw mass spectrometry data, peak lists, and results, supporting the findings of this study, are available in the following repositories: mouse data have been deposited in the ProteomeXchange Consortium via the PRIDE partner repository under the dataset identifier PXD059665 [Reviewer account details: Username: reviewer_pxd059665@ebi.ac.uk; Password: esFeXNYWrN6p]. Human data are available in MassIVE under the accession code MSV000096932 [Reviewer account details: Username: MSV000096932; Password: a]. Cytoscape files were deposited in Zenodo, entry 14751342 [DOI 10.5281/zenodo.14751342 to be published]. Source data are provided with this paper.
